# Supplementary figures and images for: Universal scale-free representations in human visual cortex
Source: PLoS Comput Biol. 2025 Nov 21;21(11):e1013714. doi: 10.1371/journal.pcbi.1013714 (PMC12654933; doi:10.1371/journal.pcbi.1013714)

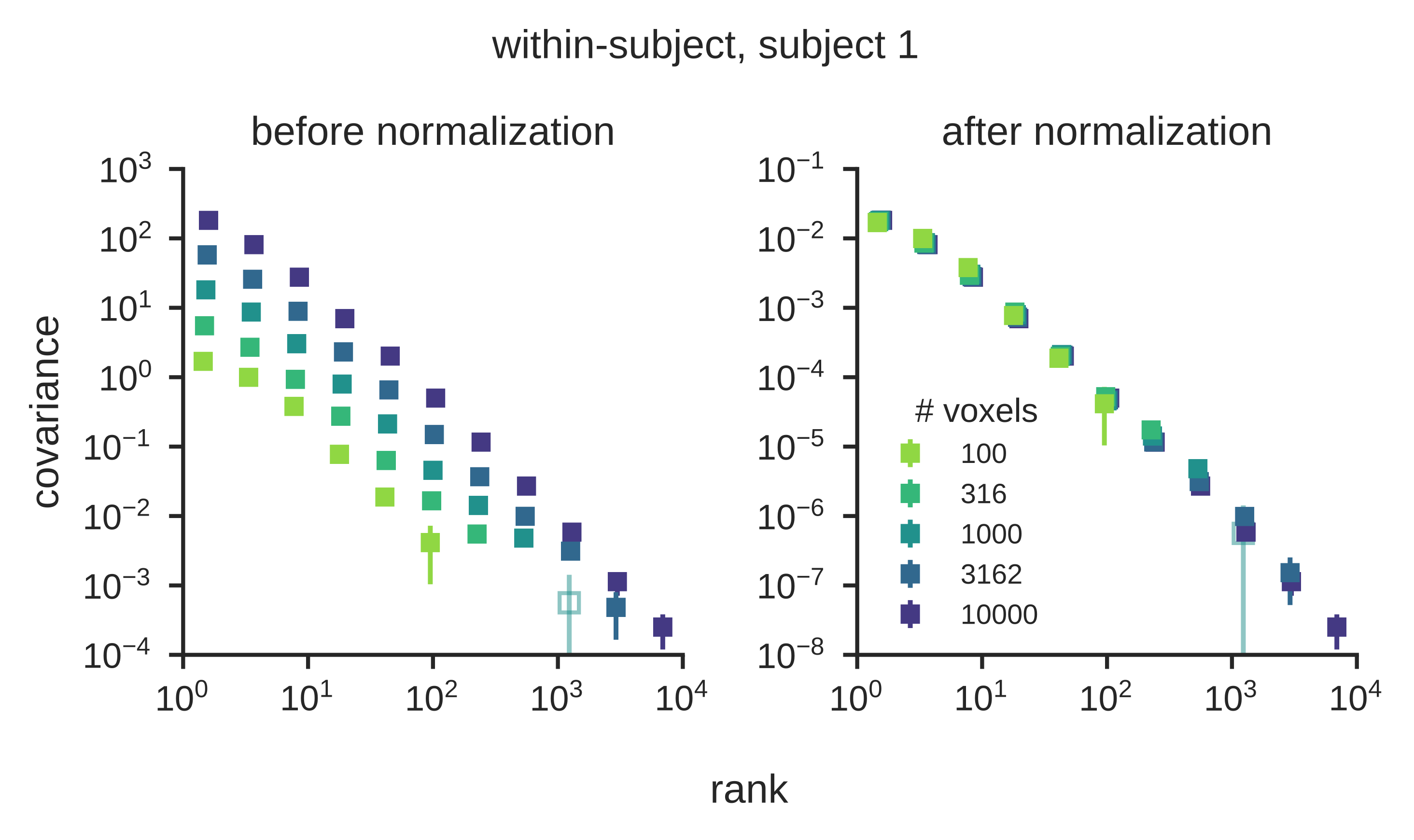

Supplement: S1 Fig — (Left) Within-subject cross-trial spectra were computed with different numbers of voxels sampled from the general region of interest. Increasing the number of voxels leads to higher variance. (Right) Normalizing the spectra by the number of voxels accounts for these differences. Open symbols denote data that are not significant at p < 0.001 (permutation tests, N = 5000). (TIFF) [file pcbi.1013714.s001.tif]

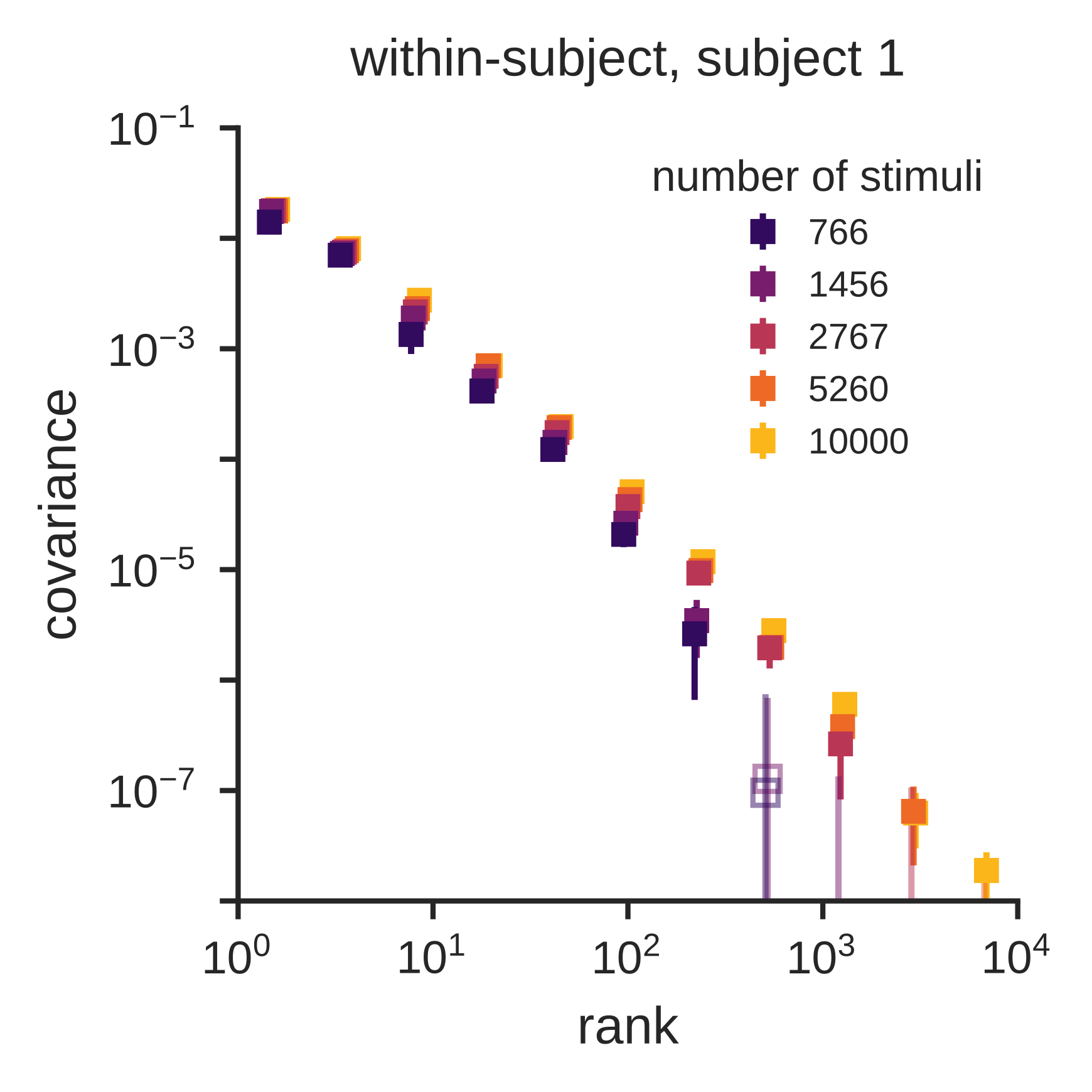

Supplement: S2 Fig — Within-individual covariance spectra for an example subject (subject 1) are computed using different numbers of stimulus images, ranging from 766 (the number of shared images seen by all participants in the experiments) to 10,000. We note that with fewer stimuli, reliable signal at high-ranks is not detectable. Open symbols denote data that are not significant at p < 0.001 (permutation tests, N = 5000). (TIFF) [file pcbi.1013714.s002.tif]

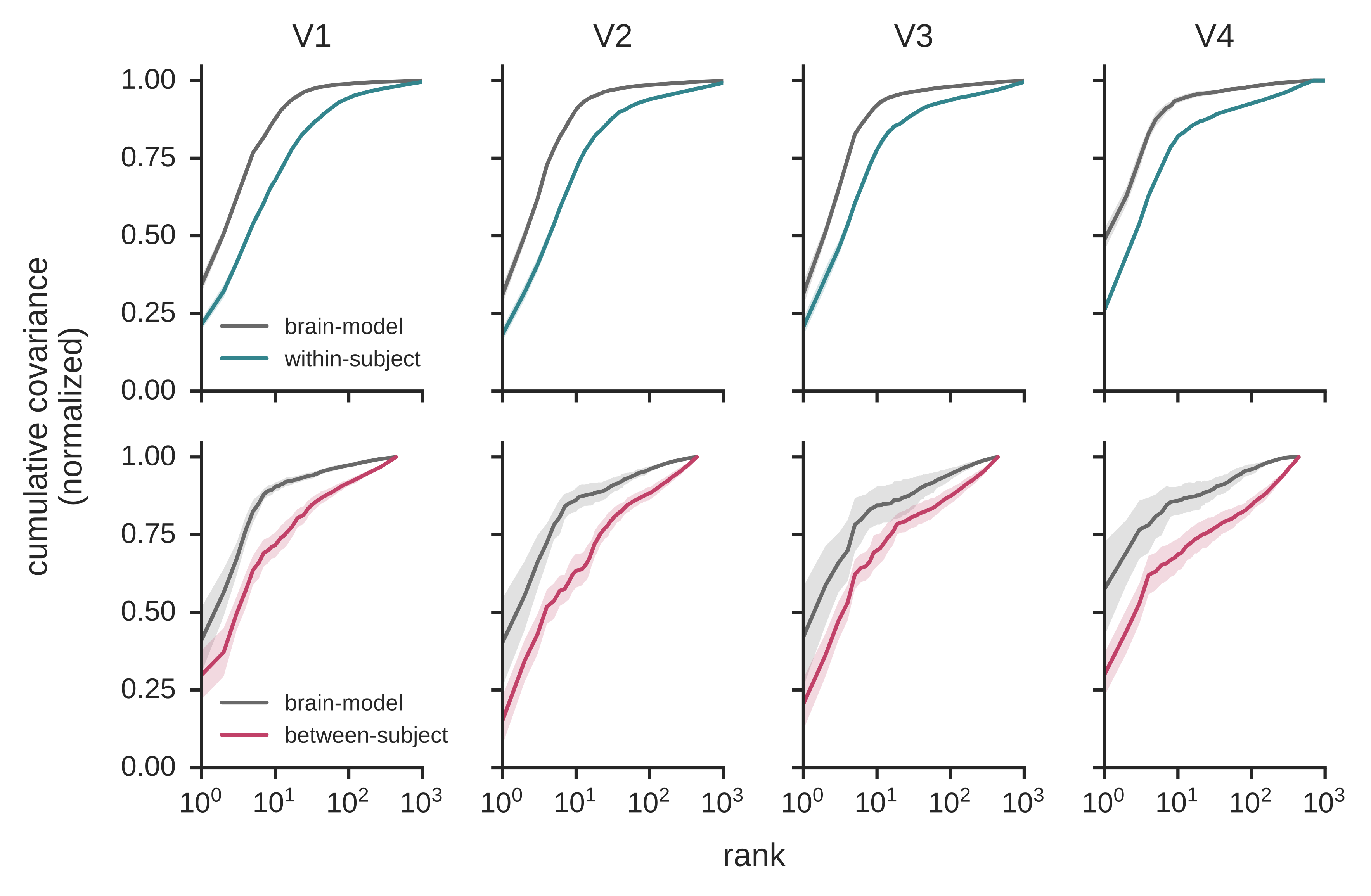

Supplement: S4 Fig — (Top) (Gray) The normalized cumulative shared variance between (i) neural responses on the first stimulus presentation and (ii) neural responses on the second stimulus presentation predicted by the Gabor model. (Green) The normalized cumulative shared variance between the neural responses to the first and second presentations of the stimuli (within-subject spectrum shown in the main manuscript). Data are shown for example subject 1. (Bottom) (Gray) The normalized cumulative shared variance between (i) neural responses for the first subject and (ii) neural responses for the second subject predicted by the Gabor model. (Red) The normalized cumulative shared variance between the neural responses of the first and second subjects (between-subject spectrum shown in the main manuscript). In all regions from V1 through V4, the Gabor model is consistently lower-dimensional than the neural data, as demonstrated by the more rapid increase in cumulative variance. Error bands denote standard deviations across 8 folds of cross-validation. Note that the between-subject analysis (bottom) uses 10 times fewer stimuli than the within-subject analysis (top) and thus has larger error bands. (TIFF) [file pcbi.1013714.s004.tif]

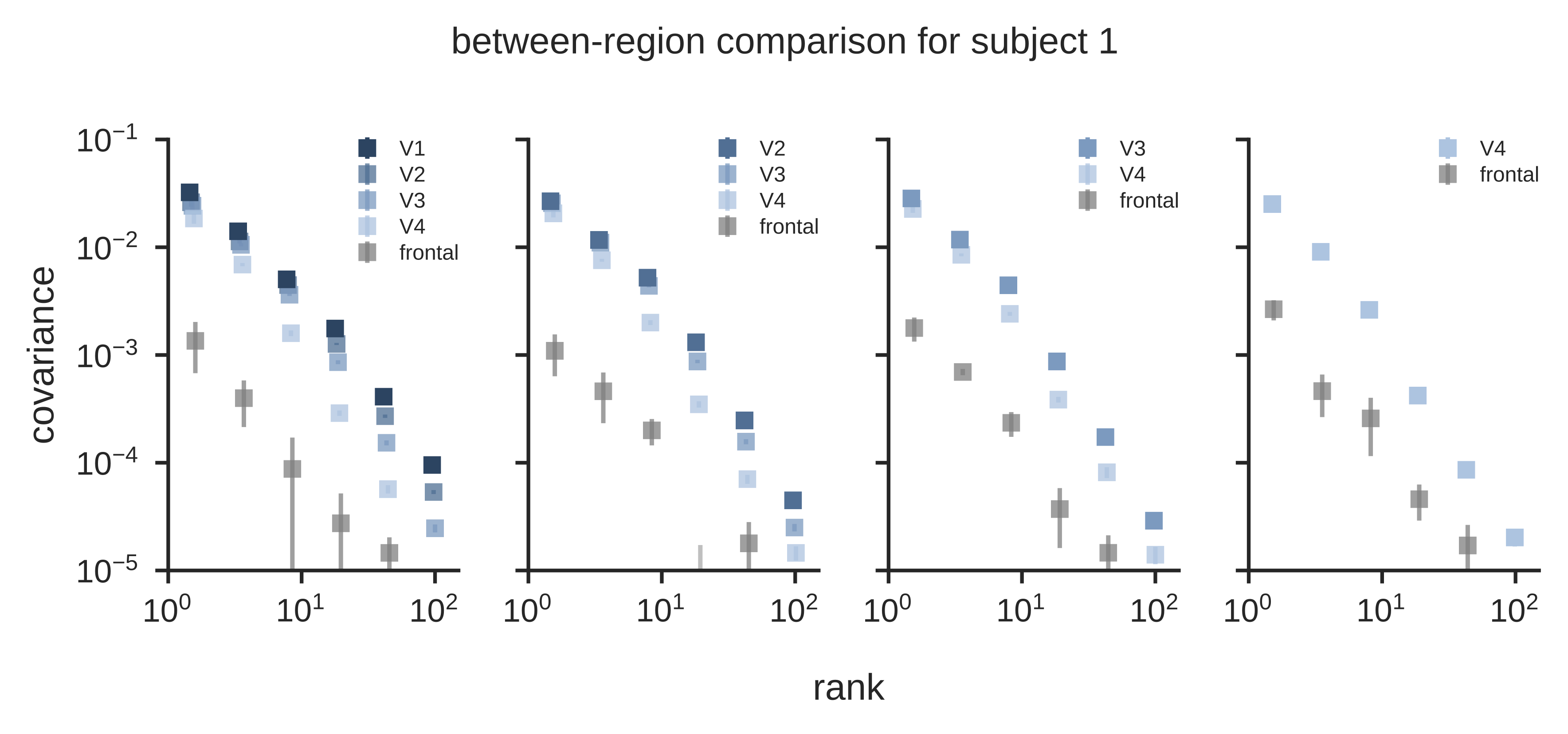

Supplement: S5 Fig — Between-region covariance spectra for visual cortex regions V1 to V4 and a large frontal region containing voxels that are not modulated by visual stimuli. All pairwise comparisons between V1, V2, V3, V4 and this frontal region are shown. The systematic decrease in between-region covariance from V1-V1 to V1-V4 recapitulates the sequential processing of information from V1 to V4. Open symbols denote data that are not significant at p < 0.001 (permutation tests, N = 5000). (TIFF) [file pcbi.1013714.s005.tif]

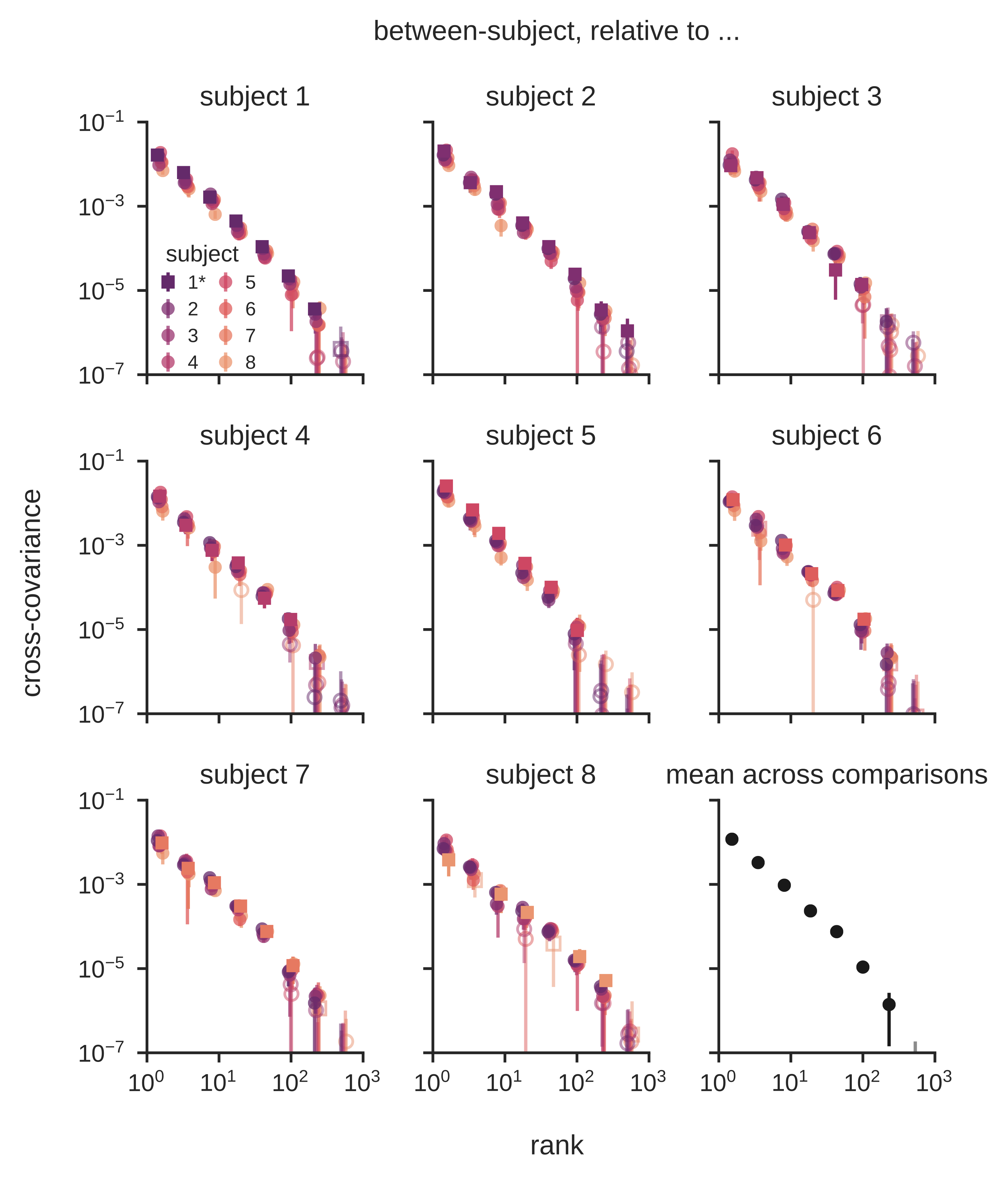

Supplement: S6 Fig — These plots show the same analysis as in the upper right panel of Fig 2 but with each subject treated as the reference subject. The last panel shows the average between-subject spectrum across all (82)=28 pairs of comparisons. Open symbols denote data that are not significant at p < 0.001 (permutation tests, N = 5000). (TIFF) [file pcbi.1013714.s006.tif]

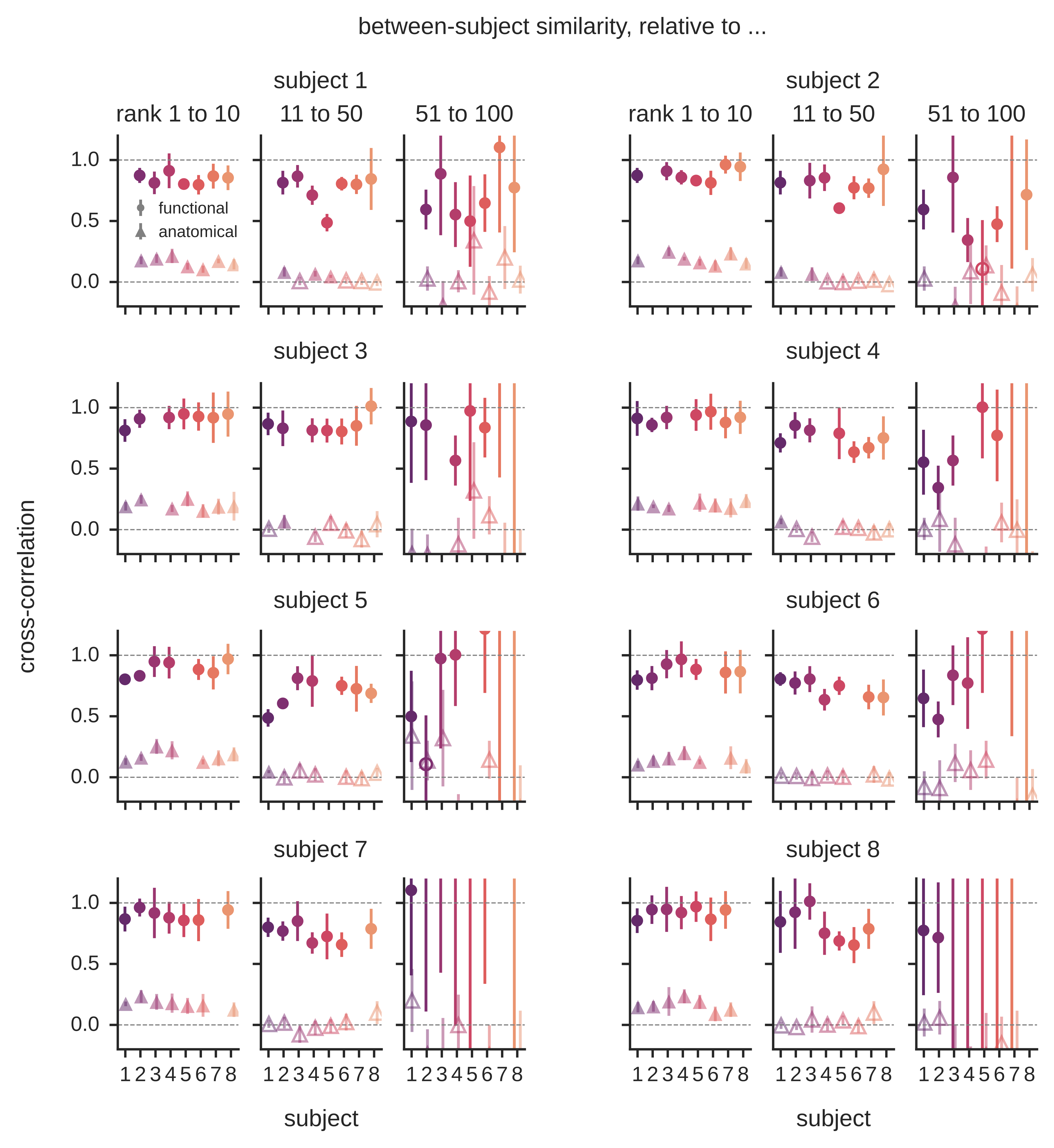

Supplement: S7 Fig — These plot shows the same analysis as in Fig 4 but with each subject treated as the reference subject. (TIFF) [file pcbi.1013714.s007.tif]

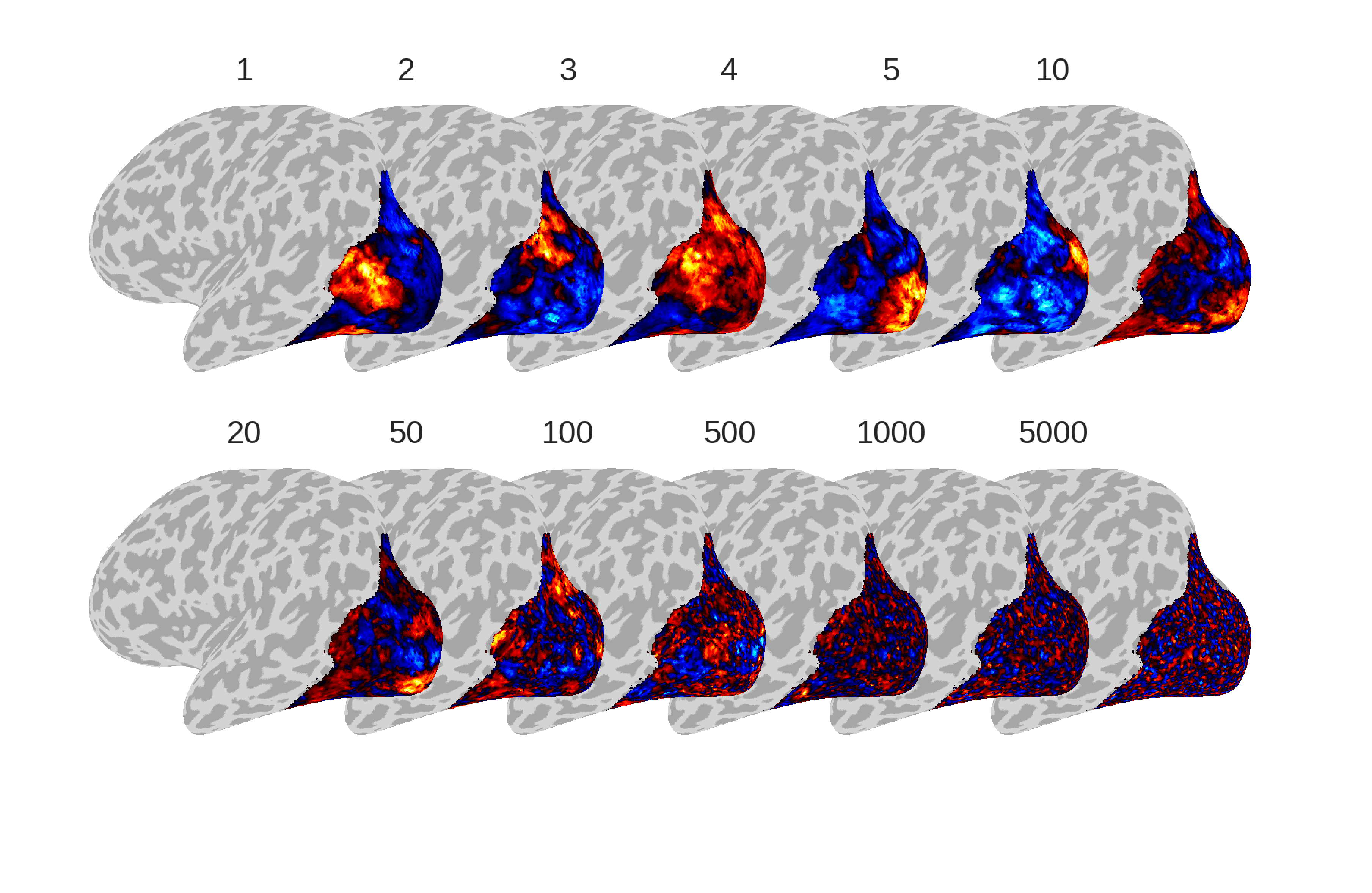

Supplement: S8 Fig — obtained from our within-subject cross-decomposition analysis for subject 1 displayed on the cortical surface. The typical spatial scale decreases monotonically with rank. (TIFF) [file pcbi.1013714.s008.tif]

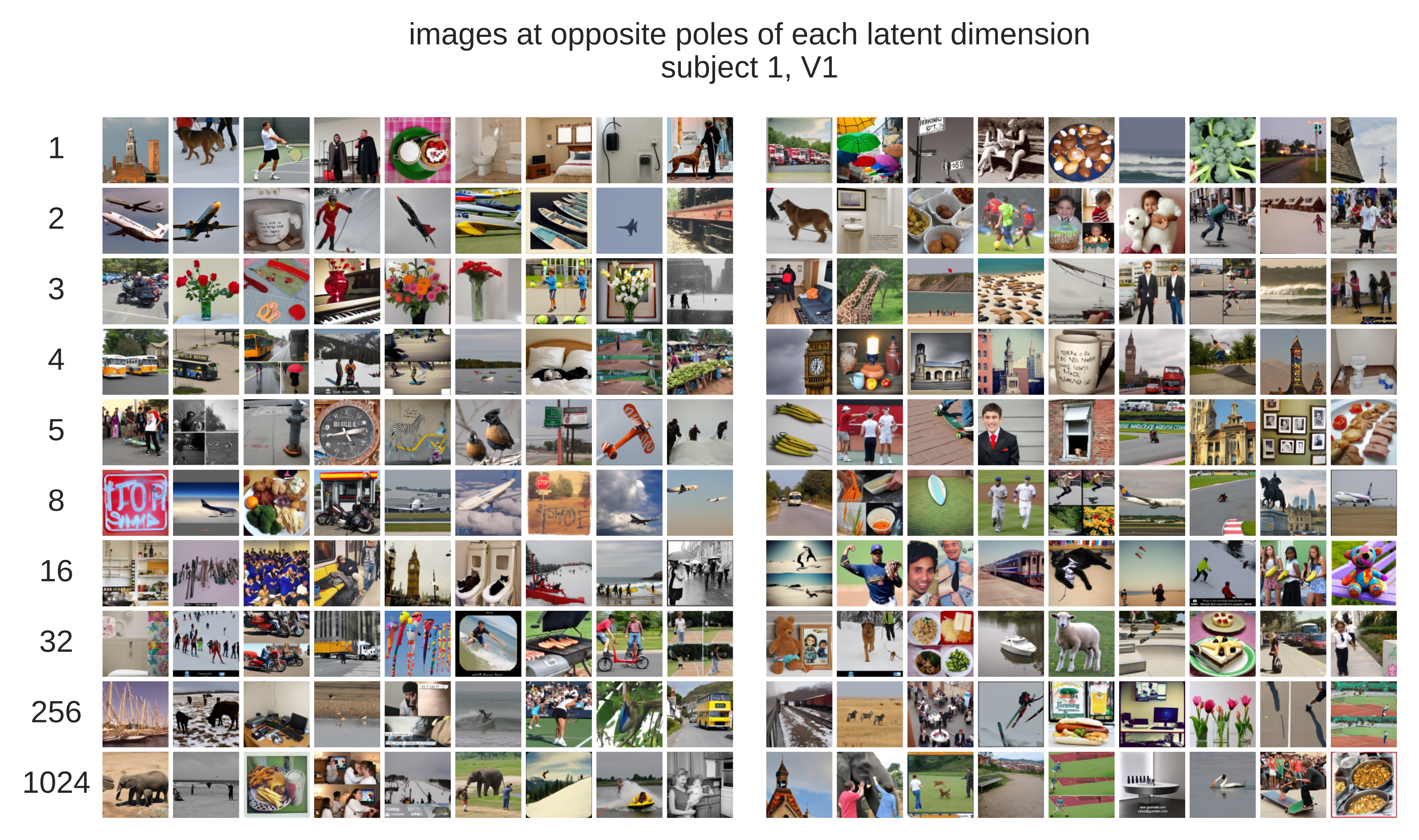

Supplement: S9 Fig — To interpret the latent dimensions of primary visual cortex (V1) responses to natural images, we visualize the images from the dataset that have the most positive (left) and most negative (right) projections on the left singular vectors extracted from within-subject cross-decomposition for an example subject (subject 1). We show images corresponding to the first few latent dimensions (1-5) followed by a small sample of high-rank dimensions (8, 16, 32, 256, 1024) throughout the spectrum. We find that the second latent dimension appears sensitive to airplanes/trains while the third latent dimension is activated strongly by flowers in vases, and the fourth is activated by clocktower-like buildings. However, we expect that these patterns are driven by low-level features such as spatial orientation that covary strongly with these semantic features in these images. Meanwhile, the high-rank dimensions show no interpretable patterns. Due to restrictive licensing, the original images have been replaced in this visualization with semantically similar images generated from the diffusion model CommonCanvas-XL-C based on human-generated captions [50]. Note that the perceptual details of these synthesized images differ from the original images. (TIFF) [file pcbi.1013714.s009.tif]

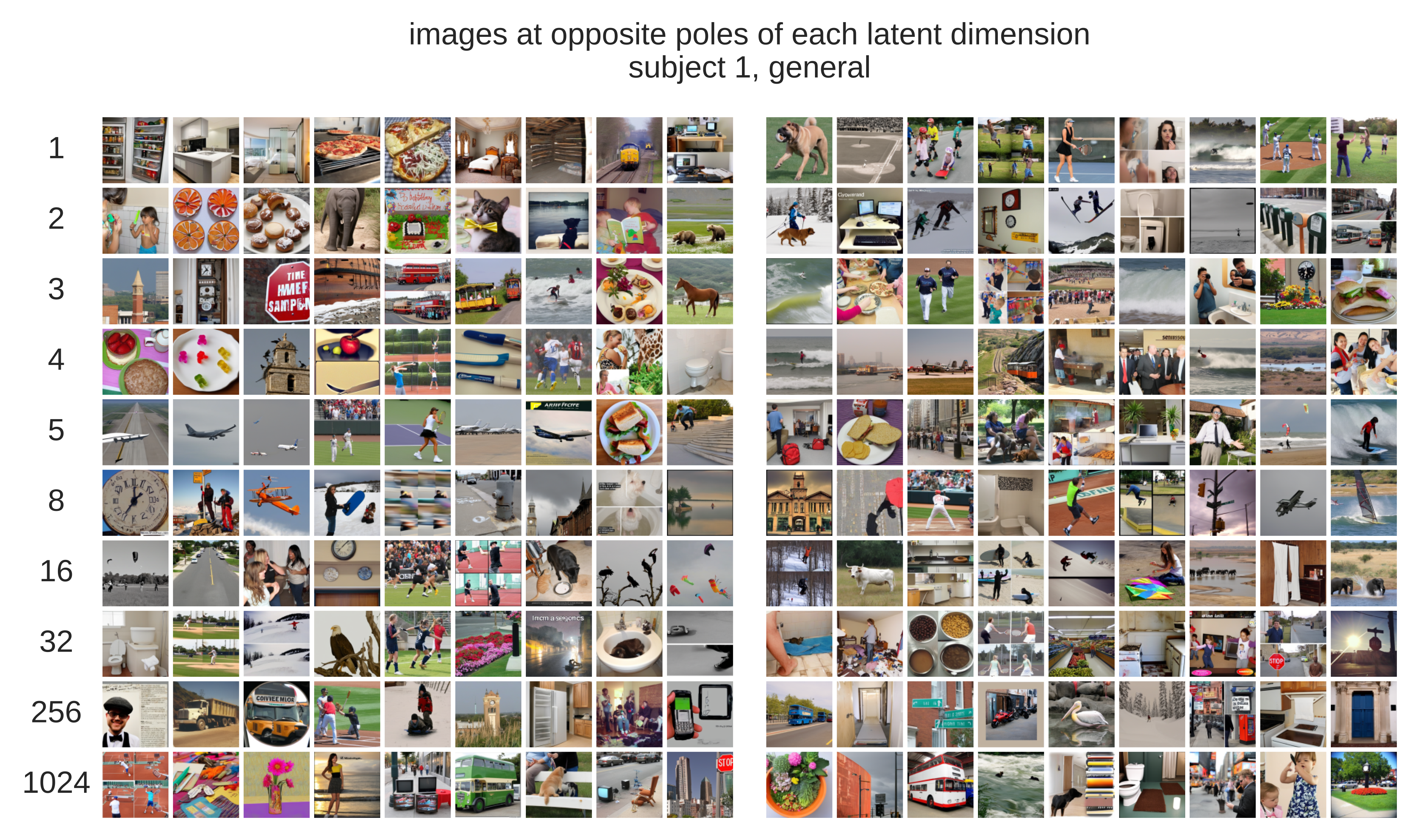

Supplement: S10 Fig — We repeat the analysis described in S9 Fig, using the “general” visually responsive region instead of primary visual cortex (V1). Here, the first latent dimension appears to be correlated with animacy (S13 Fig, top, first pair of bars): it separates images with people (right) from images without people (left). The high-rank dimensions again show no interpretable patterns. Due to restrictive licensing, the original images have been replaced in this visualization with semantically similar images generated from the diffusion model CommonCanvas-XL-C based on human-generated captions [50]. Note that the perceptual details of these synthesized images differ from the original images. (TIFF) [file pcbi.1013714.s010.tif]

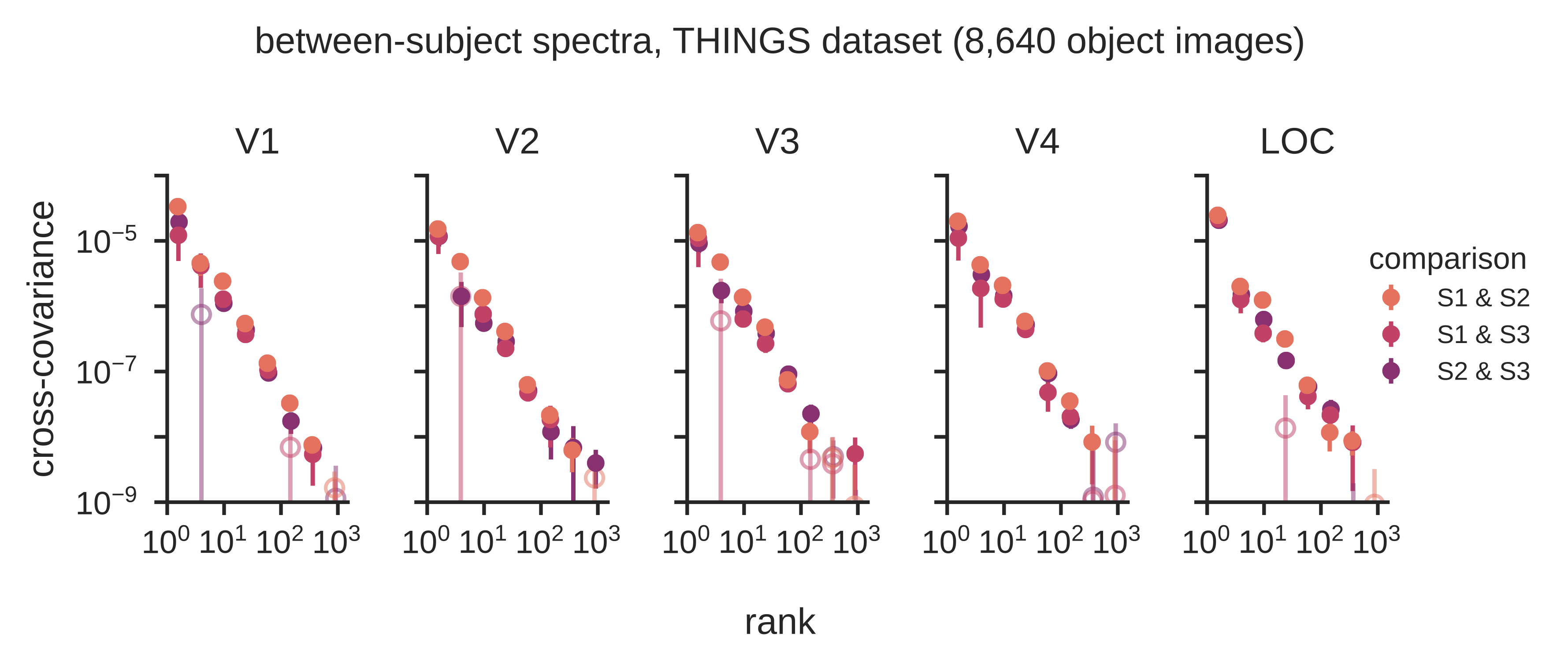

Supplement: S11 Fig — These plots show between-subject covariance spectra of visual cortex responses in V1 through V4 and object-selective lateral occipital complex (LOC) from the THINGS-data fMRI dataset [16]. All spectra are normalized to account for differences in the number of voxels across participants and averaged within bins of exponentially increasing width and across 8 folds of cross-validation. Error bars denote standard deviations across these 8 folds. Open symbols denote data that are not significant at p < 0.001 (permutation tests, N = 5000). (TIFF) [file pcbi.1013714.s011.tif]

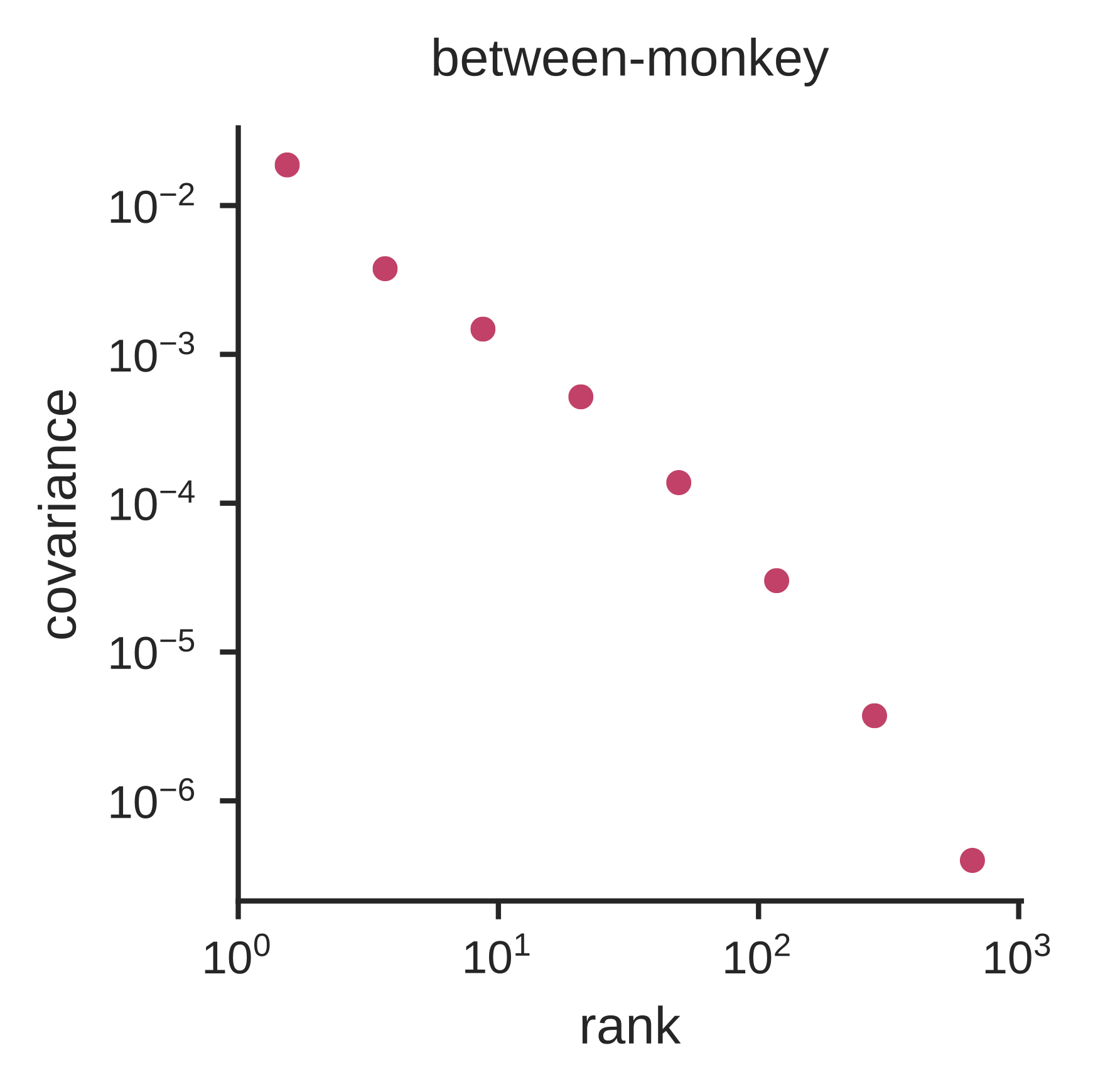

Supplement: S12 Fig — This plot shows the between-monkey covariance spectrum of visual cortex responses in V1, V4, and IT from the THINGS ventral stream spiking dataset [17]. The spectrum is averaged within bins of exponentially increasing width and across 8 folds of cross-validation. Error bars denote standard deviations across these 8 folds. Open symbols denote data that are not significant at p < 0.001 (permutation tests, N = 5000). Note that the error bars are small and not visible. This is because this dataset contains twenty times more stimuli than channels, and thus, the covariance estimates are highly consistent across splits of the data. (TIFF) [file pcbi.1013714.s012.tif]

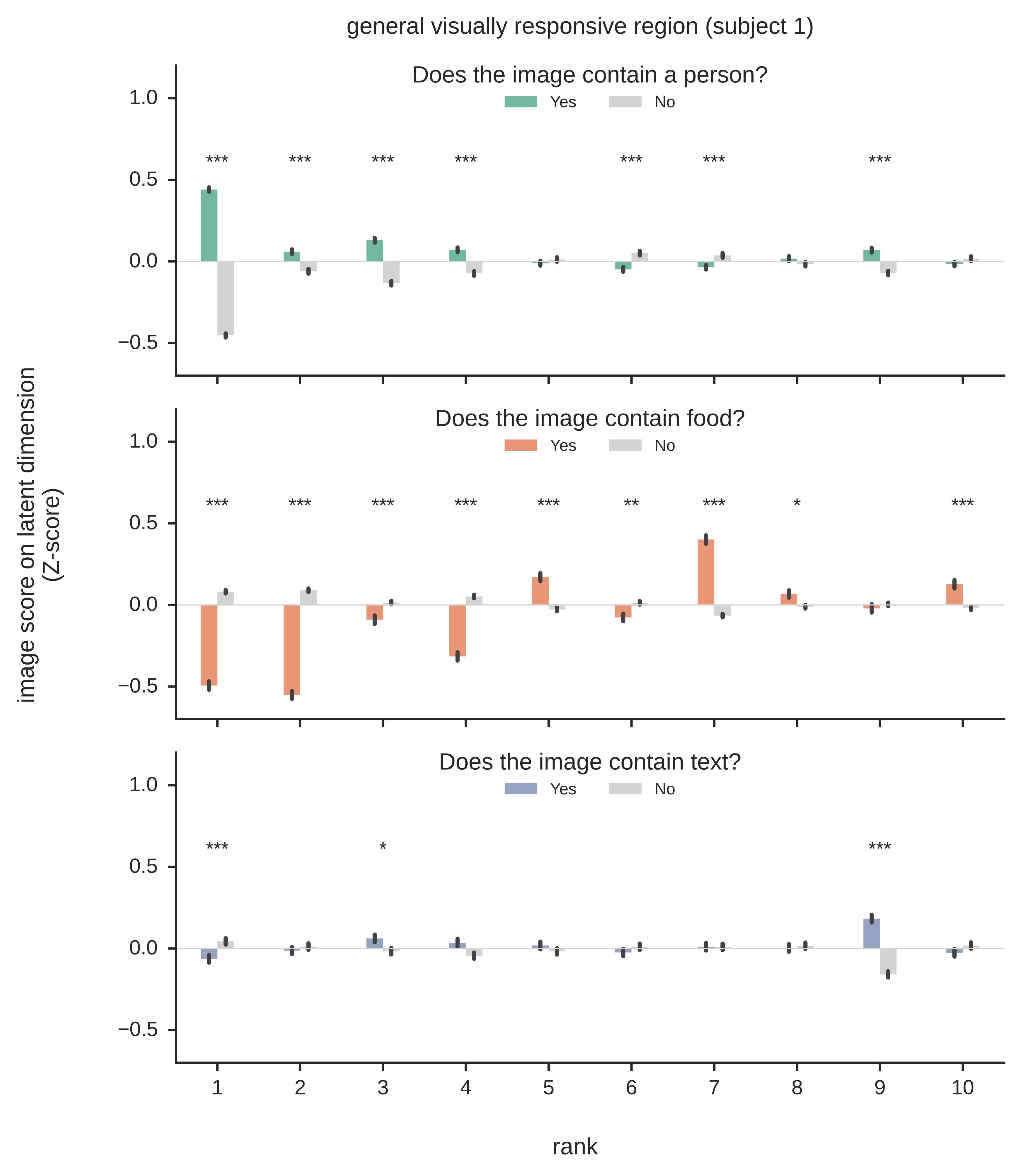

Supplement: S13 Fig — Information about the presence of people (top), food (middle) and text (bottom) in an image is distributed over the first 10 latent dimensions of neural activity in the “general” visually responsive region of cortex, identified through within-subject cross-decomposition in an example subject (subject 1). Images that either contain (colored) or do not contain (gray) these object categories are projected onto each of these dimensions and these score distributions were compared using Mann-Whitney U tests to identify significant differences (*, p < 0.01; **, p < 0.001; ***, p < 0.0001). Bars and error bars indicate the means of the score distributions and their standard errors. (TIFF) [file pcbi.1013714.s013.tif]

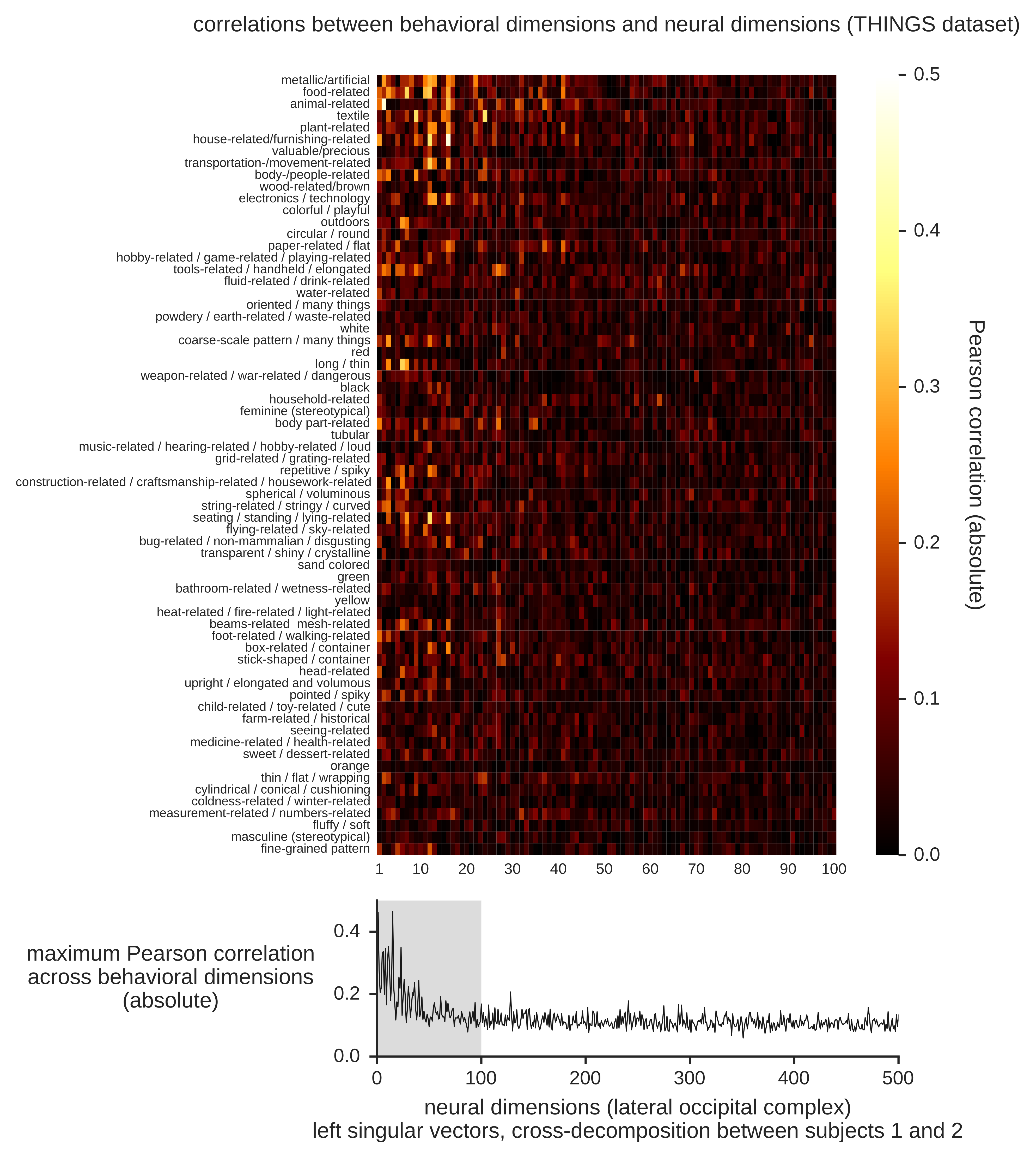

Supplement: S14 Fig — We perform between-subject cross-decomposition of the neural responses in the lateral occipital complex (LOC) from subjects 1 and 2 and project all the images onto the latent dimensions from subject 1. Then, we average these projections across all images within each of the 720 object categories and correlate each of these neural dimensions with each of the 66 interpretable dimensions that capture human object similarity judgments in a large-scale behavioral experiment [16]. (Top) We find correlations between the neural and behavioral dimensions but no evidence for a one-to-one correspondence between these dimensions. (Bottom) For each neural dimension, we compute the maximum absolute Pearson correlation with all behavioral dimensions and find that the correlations between neural and behavioral dimensions are strongest in the low-rank dimensions and fall off rapidly. (TIFF) [file pcbi.1013714.s014.tif]
