# Supplementary material for: Universal scale-free representations in human visual cortex
Source: PLoS Comput Biol. 2025 Nov 21;21(11):e1013714. doi: 10.1371/journal.pcbi.1013714 (PMC12654933; doi:10.1371/journal.pcbi.1013714)
Supplement: Attachment — Submitted filename: Response to Reviewers.pdf [file pcbi.1013714.s015.pdf]

Response to reviewer comments

Raj Magesh Gauthaman[1], **Brice Ménard**[2,1,3], **Michael F. Bonner**[1]
[1]Department of Cognitive Science, [2]Department of Physics and Astronomy
Johns Hopkins University, Baltimore, MD
[3]Santa Fe Institute, Santa Fe, NM
{rgautha1, bmenard1, mfbonner}@jh.edu

1 Reviewer #1

> This paper by Raj and colleagues analyzes the structure of visual (fMRI) representations and show that the variance decays as a power law. This suggests that there is no systematic scale that dominates representations (information is distributed across dimensions). The study goes beyond prior approaches that employed statistical methods like PCA and ICA in characterizing the statistical structure (and not focusing on the specific visual feature encodings supported by the dimensions). Additional key results also demonstrate that A) the scale-free structure is remarkably preserved across individuals (Figure 2,4), B) is not observed in non-visual frontal regions (when compared between-subjects, Figure 3), and C) is present across the visual hierarchy (until V4, Figure 5,6). Overall, the paper is well-written, with clear results and a fair consideration of alternative explanations, including exponential decay and low-dimensional models.

Thank you!

> I have three main concerns regarding the strong claim of "universality" in scale-free representations of the visual cortex.
>
> 1. Since the study does not use the full NSD stimulus set (due to cross-subject comparisons), it would be valuable to test whether these findings replicate in other large-scale fMRI datasets, such as THINGS or BOLD5000v2. Expanding the analysis to additional datasets would strengthen the claims about the universality of the scale-free structure and confirm that the results are not specific to the NSD dataset. There is a small concern of non-independence between train and test sets in NSD (as noted by Shirakawa et al. (2024)). This issue could be completely mitigated by verifying whether the same statistical properties hold in an entirely separate dataset. A cross-dataset validation would provide stronger evidence that the observed scale-free structure is a robust feature of visual cortex organization rather than an artifact of dataset-specific factors.

We agree with the referee that extending the analysis to a different large-scale dataset of cortical responses to natural images would strengthen our results and the claim of universality. As the reviewer suggested, we have repeated our analyses on the THINGS-data fMRI dataset (Hebart et al., 2023), in which 3 participants viewed 8,640 images of objects centered on a naturalistic background, with 12 images from each of 720 object categories ("concepts"). This number of unique stimuli is comparable in scale to the Natural Scenes Dataset (~10,000 images per participant) (Allen et al., 2021). However, in the THINGS fMRI dataset, each image is seen only once by each participant, making it impossible to run our within-subject cross-decomposition analysis, which relies on having repeated measurements of cortical responses to the same stimuli. However, the same 8,640 images are seen by all three participants in the dataset, allowing us to run our between-subject cross-decomposition analysis with higher statistical power than in the NSD,

where only ~1,000 images are shared across participants. As the between-subject analyses are a more stringent test than the within-subject analyses, this makes for a compelling replication of our results in the NSD dataset.

Here, we analyze regions V1 through V4 identified through population receptive field (pRF) mapping to provide a direct comparison to the NSD where we analyzed the same four visual regions (Figure 5, right), as well as the object-selective lateral occipital complex (LOC).

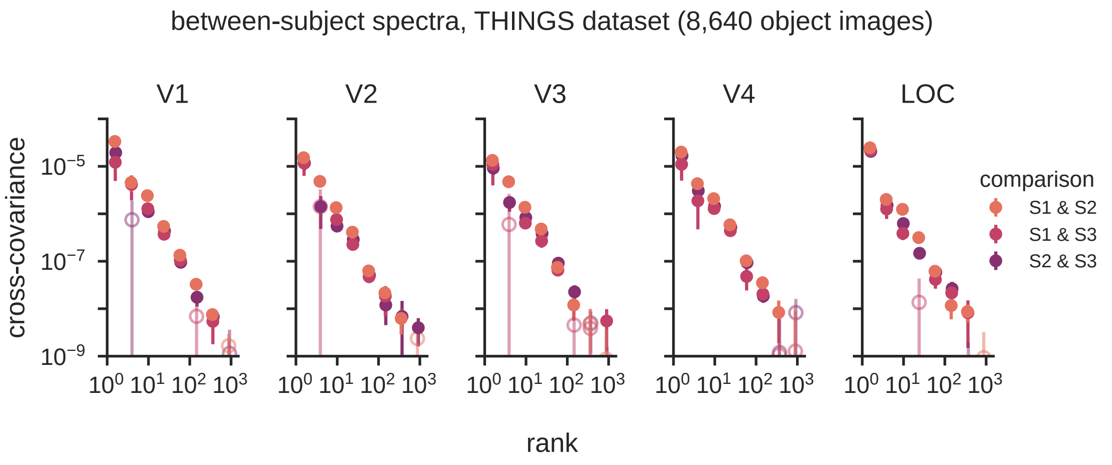

Figure 1: **Power-law spectra of between-subject covariance in the THINGS dataset of fMRI responses to object images.** This plot shows between-subject covariance spectra of visual cortex responses in V1 through V4 and object-selective lateral occipital complex (LOC) from the THINGS-data fMRI dataset (Hebart et al., 2023). All spectra are normalized to account for differences in the number of voxels across participants and averaged within bins of exponentially increasing width and across 8 folds of cross-validation. Error bars denote standard deviations across these 8 folds. Open symbols denote data that are not significant at $p < 0.001$ (permutation tests, $N = 5000$).

Our results show that there is a high-dimensional spectrum of shared variance shared between subjects in each of these regions of interest (Figure 1), corroborating our findings in the NSD data. We are not limited by the number of shared stimuli but instead by the number of voxels (~1,000) in each of these regions of interest. These results in a new dataset confirm that the scale-free population covariance structure is a robust feature of visual cortex representations and not an artifact of the NSD dataset.

2. At some stage, neural representations must become low-dimensional to align with behaviorally relevant features. A key question is whether the scale-free structure observed here is specific to early and mid-level visual areas or if it extends to higher-level visual regions. Since the study primarily focuses on early-to-mid-level regions, it's unclear whether the same pattern would hold in later stages of processing. One way to test this is by analyzing artificial neural networks (ANNs). If the findings generalize, we would expect early and mid-level layers to exhibit scale-free structure, while higher layers—where abstract, behaviorally relevant representations emerge—would not. Testing this would provide stronger support for claims of universality and clarify whether this pattern is truly a fundamental property of the visual cortex or just a characteristic of its early processing stages.

This is a topic that we are very interested in (and pursuing as a future direction). However, we believe it remains an open question whether higher-level cognitive regions rely on low-dimensional versus high-dimensional representational formats. While many have speculated about the importance of low-dimensional structure in association and motor cortices, there are also compelling arguments and empirical evidence suggesting that previous findings of low-dimensionality may reflect the limited complexity of the stimuli and behavioral tasks in conventional neuroscience studies (Sorscher et al., 2022; Yan et al., 2020). Furthermore, there are compelling theoretical arguments and empirical evidence suggesting that high-level cognitive regions rely on high-dimensional representations to support flexible behaviors (Fusi et al., 2016; Posani et al., 2024; Rigotti et al., 2013).

To empirically investigate this question in the data examined here, we split the NSD general ROI in half along the posterior-anterior axis to create posterior and anterior visual ROIs with an equal number of voxels. We then analyzed the within-subject covariance spectra in these regions. We found that the reliable stimulus-related information in the higher-level anterior region is distributed over many hundreds of latent dimensions, much like the spectrum observed in the lower-level posterior region (Figure 2). Thus, the scale-free spectrum observed here appears to be a general property of both lower-level and higher-level regions of the visual system.

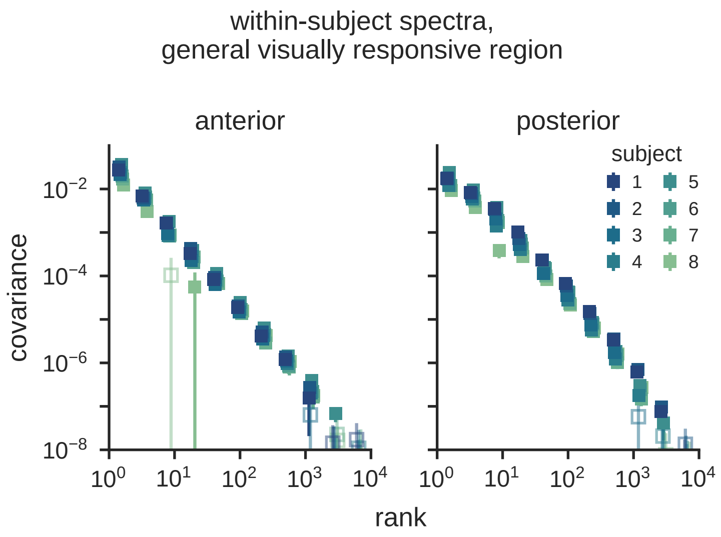

Figure 2: **Within-subject covariance spectra in anterior (high-level) and posterior (low-level) visual cortex.** The "general" visually responsive region from the Natural Scenes fMRI dataset (Allen et al., 2021) was divided into anterior and posterior sections containing the same number of voxels. All spectra are normalized to account for differences in the number of voxels across participants and averaged within bins of exponentially increasing width and across 8 folds of cross-validation. Error bars denote standard deviations across these 8 folds. Open symbols denote data that are not significant at $p < 0.001$ (permutation tests, $N = 5000$).

Finally, we want to note that while we are interested in the question of how dimensionality changes across the hierarchy of artificial neural networks, we believe that this question falls too far out of scope for the current manuscript, which focuses on human brain activity. We thus prefer to leave extensions of this work to artificial neural networks as a future direction.

3. How much of the observed result is because of fMRI-related smoothing? Since fMRI signals aggregate activity across thousands of neurons within each voxel, it's possible that this averaging inherently produces a power-law appearance in the variance spectrum. The analyses of frontal regions addresses this problem to some degree. Al alternate strategy could be to conduct simulations to test the conditions under which scale-free structure could emerge simply from averaging over many independent neural responses. Another option is compare the same results against publicly available monkey Utah array recordings (like the Many Monkeys dataset from the DiCarlo lab).

Although heavy-tailed distributions can arise from a procedure that takes the product of many random positive numbers, they do not arise due to the simple averaging of many numbers, which instead yields a normal distribution by the central limit theorem. As an empirical confirmation that the spectrum observed in our work is not simply due to fMRI-related smoothing, we used our cross-decomposition method to re-analyze a dataset of single-neuron responses to natural images in mouse V1 measured using calcium imaging (Stringer et al., 2019). We again observed a power-law spectra, replicating the findings from the original work by Stringer et al. but with a more rigorous cross-validation approach. This finding using single-unit data from mice demonstrates that the power-law spectra observed in our work is not an artifact of fMRI-related smoothing.

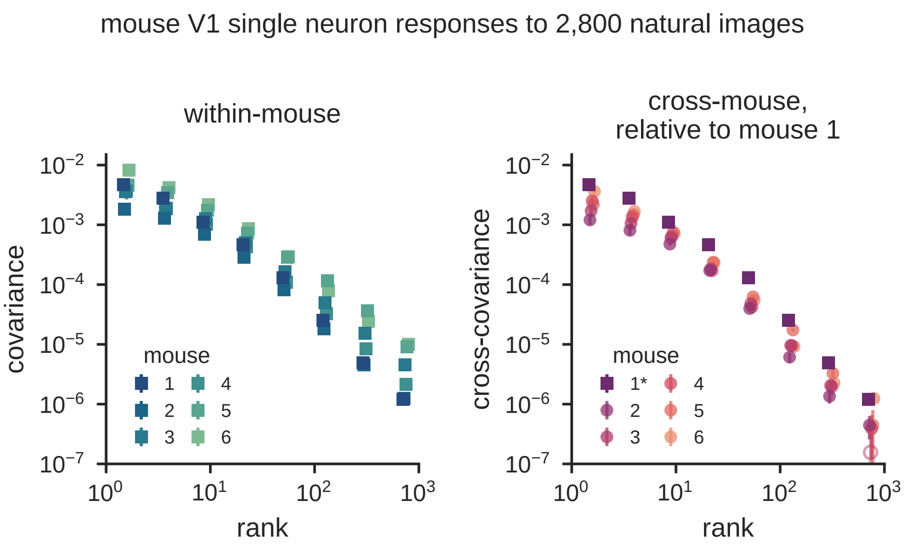

Figure 3: **Within- and between-individual power-law covariance spectra in mouse V1 single neuron responses to natural images.** Within-mouse (left) and between-mouse (right) covariance spectra computed for mice measuring the spectrum of reliable variance in neural responses. All spectra are normalized to account for differences in the number of voxels across participants and averaged within bins of exponentially increasing width and across 8 folds of cross-validation. Error bars denote standard deviations across these 8 folds. Open symbols denote data that are not significant at $p < 0.001$ (permutation tests, $N = 5000$).

2 Reviewer #2

This manuscript studies the dimensionality of visual representations across subjects in responses to naturalistic images. Rather than seek to understand what labeled, computed, or otherwise described visual features or dimensions of features drive responses in different functionally or anatomically labeled areas, the authors seek to understand the distribution of dimensions of the visual representation in terms of (1) how many dimensions of visual responses are shared across individuals, (2) how much variance each (range) of those dimensions explains. They find that the variance explained by stimulus-driven dimensions of visual features decreases in a way consistent with scale-free representation of visual information.

This paper is technically sound (tho with some omissions, see below), and takes a somewhat novel approach to understanding visual representations. My main issue with this manuscript is that I have a hard time understanding what the results add to our understanding of the visual system. I do not want to disparage the approach for being unfamiliar to me, and the analyses seem well-constructed and valid. However, I think the authors need to do a better job explaining how their results connect to extant literature, and need to do a better job explaining their dependent measures and how to interpret them.

Thank you. As detailed in our responses below, we have performed new analyses and made edits to strengthen the manuscript and clarify how this work advances our understanding of the visual system.

2.1 Major points

It is not clear to me that the observation of a scale-free representation is unexpected or otherwise surprising. Should we expect representation of visual information to be scale-free, either a priori (based on first principles) or based on other work? It sound from the literature cited as if the weight of available evidence - prior to this paper - suggests a scale-free representation. So what does this manuscript add to the insights derived from other papers? It would help if the authors framed the implications of their results in a way that did not use the words “scale-free” as much. The repetition of this phrase makes the results

seem abstruse and/or tightly linked to this specific analysis. What were meaningful alternatives that may have been encountered here, and what would they have meant?

We thank the reviewer for raising this question. It made us realize that we needed to explain our key theoretical contributions more directly and clearly. Indeed, our work makes important new contributions, and our findings have broad implications for theories of the human visual system and for the methods that are used to study its representations. We have now made edits to the Introduction and Discussion to better emphasize these points (lines 57-60, 65-67, 83-86, 307-318, 336-342, 348-351). To summarize, our contributions are the following:

Novelty. Our work is the first demonstration of the power-law structure underlying human cortical representations of natural images. In the systems neuroscience literature, Stringer et al. (2019) demonstrated the presence of scale-free visual representations in mouse V1. However, it remained unknown whether **(1)** the same power-law structure spanning several orders of magnitude would also be found in the human visual system, **(2)** whether it would be found in higher-level visual regions beyond V1, **(3)** whether it could be reliably detected in fMRI data, and **(4)** whether the underlying dimensions were shared across individuals or instead reflected idiosyncratic aspects of each subject's brain activity patterns. Our findings rigorously answer all these questions for the first time.

Theoretical implications. Our results contrast strongly with previous literature on cortical representations in visual cognitive neuroscience. Most work has found a relatively small number of informative dimensions in cortical data, typically ranging from ~4 to ~87 (Hebart et al., 2023; Huth et al., 2012; Khosla et al., 2022; Lehky et al., 2014; Tarhan & Konkle, 2020), and moreover, an influential theory has argued that the goal of the visual hierarchy is to compress sensory inputs onto a low-dimensional manifold that is robust to identity-preserving perturbations (Fusi et al., 2016; Lehky & Tanaka, 2016; Lehky et al., 2014). It was thus not at all obvious from previous work that the image representations of human visual cortex would be fundamentally high-dimensional (spanning at least thousands of dimensions), and in fact, some previous work had made the opposite prediction (Lehky & Tanaka, 2016). Furthermore, since the spectrum follows a power law up to the limit of detectability without any sign of a low-dimensional cut-off, we anticipate that collecting more data will reveal even more reliable dimensions. This suggests that contrary to the predominant approach in the field, we will never fully understand these representations by attempting to catalog and interpret each of their constituent dimensions (Hebart et al., 2023; Khosla et al., 2022).

Methodological implications. Our findings have broad implications for representational modeling because they show that conventional approaches, such as RSA, provide an extremely low-dimensional view and are insensitive to information beyond the first ~10 principal components of neural activity. Unless one believes that each region of the human brain can be fully described by just 10 dimensions, then it will be crucial for future work to adopt methods that see beyond this limited view (such as the cross-decomposition method in our work). We demonstrate this important point by showing that between-subject RSA is insensitive to information beyond the first 10 principal components, while in contrast, our cross-decomposition approach reveals that individuals share representations spanning hundreds of dimensions.

The authors need to do a better job connecting their results to the extant literature on dimensions of visual representation. They neither demonstrate nor speculate on what visual information may be in either the low-rank or the high-rank dimensions. There is a substantial literature (which the authors cite) on what the first few dimensions (generally up to 3-4, but as many as tens in some cases) of visual representation may contain, e.g. an inanimate/animate distinction, a fovea-periphery distinction, a real-world-size distinction, differences between spiky and smooth stimuli and other object properties, and more - Bao et al., 2020; Contier et al., 2024; Hebart et al., 2023; Huth et al., 2012; Tarhan & Konkle, 2020). Are there any indications that the low-rank dimensions span a similar space as any of these proposed dimensions?

Within the first few latent dimensions of each region, we find selectivity for high-level properties consistent with previous work (Figure 4). For example, the 1st dimension of responses in the "general' ' visually responsive ROI appears to capture animacy (Figure 5). Similarly, the 1st dimension of responses in face-selective cortex separates faces and non-faces (Figure 6), and the 2nd dimension of responses in place-selective cortex separates outdoor and indoor scenes (Figure 7).

However, we want to emphasize that caution is needed when trying to derive intuitive interpretations from the visual inspection of representational dimensions. Any meaning that we intuitively assign to these dimensions could be

confounded with less-salient low-, mid- or even high-level visual features. For example, in Figure 8, we observe that the 2nd latent dimension of V1 responses appears sensitive to airplanes/trains, which one might interpret as a “transportation’ ’-related dimension (especially, if this trend were observed in some higher-level ROI). Similarly, the 3rd latent dimension is activated strongly by flowers in vases, and the 4th is activated by clocktower-like buildings. However, we do not expect V1 to represent these semantic properties of images; rather, it is likely sensitive to low-level features, such as different spatial orientations, that covary strongly with these semantic features. While it seems obvious that such confounds are the likely explanation for these V1 results, we want to note that similar and more insidious confounds could very well underlie the interpretation of higher-level regions as well.

Finally, as expected, these analyses also show that in all ROIs, higher-rank dimensions are difficult to interpret, and we caution against trying to provide semantic labels to these dimensions. Indeed, there is no *a priori* reason that every dimension of the cortical code should be semantically interpretable through traditional categories or nameable concepts. For example, textures are visually salient and crucial for various visual tasks, but their features are best characterized through a statistical approach (Portilla, 2000) rather than through high-level semantics. We hypothesize that a similar story applies to the bulk of cortical dimensions—that is, much of what visual cortex represents may be best understood statistically/computationally rather than through the intuitive interpretation of image properties.

We have now added new supplementary figures for visualizations of V1 and the general region (Figures S9, S10, and S11). These figures are referred to in lines 445-452 of the Discussion.

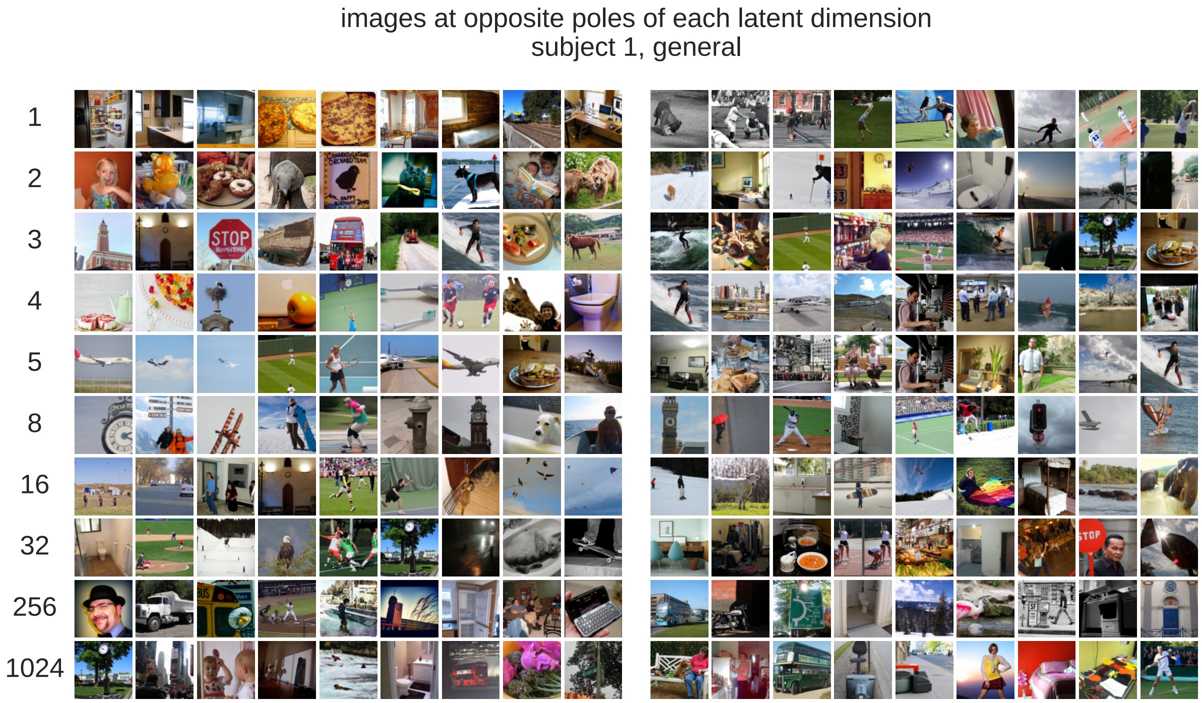

Figure 4: **Visualizing images from the dataset that strongly activate different latent dimensions of the visual cortex.** The first latent dimension appears to be correlated with animacy (Figure 5): it separates images with people (right) from images without people (left).

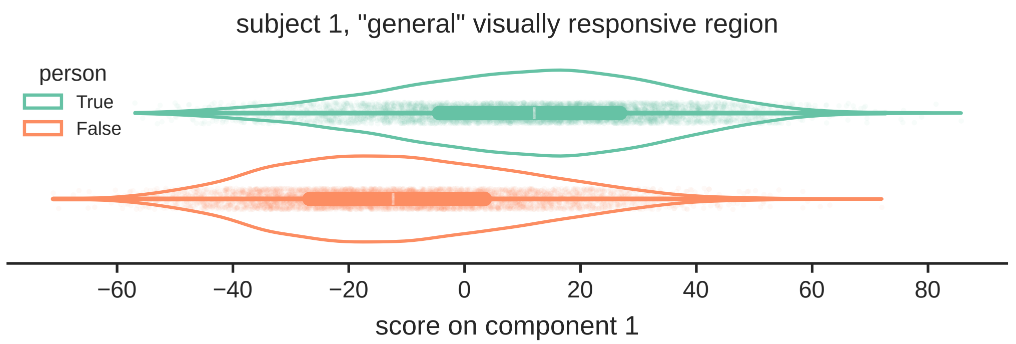

Figure 5: **The highest variance component of visual cortex responses to natural scenes encodes animacy.** Distributions of images with people (green) and without people, ordered by their projections onto the first latent dimension of visual cortex responses.

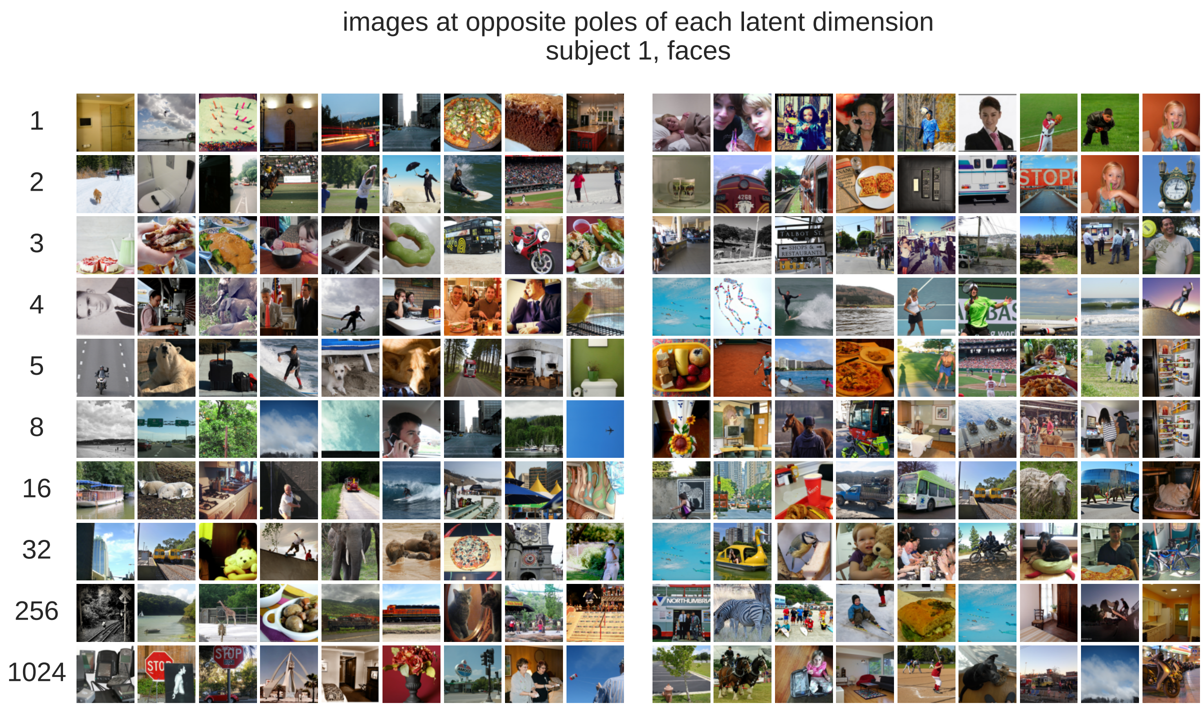

Figure 6: **Visualizing images from the dataset that strongly activate different latent dimensions of face-selective cortex.** The first latent dimension appears to separate images with faces (right) from images without faces (left).

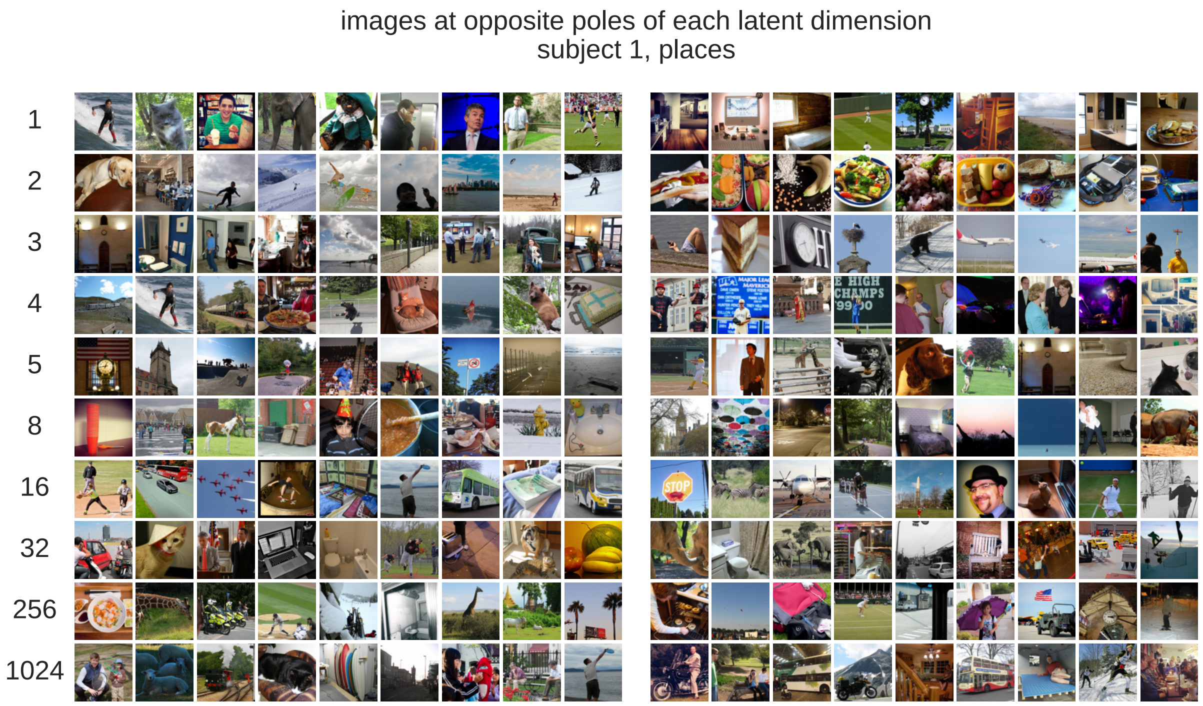

Figure 7: **Visualizing images from the dataset that strongly activate different latent dimensions of place-selective cortex.** The second latent dimension appears to separate outdoor scenes (left) from indoor scenes containing food (right).

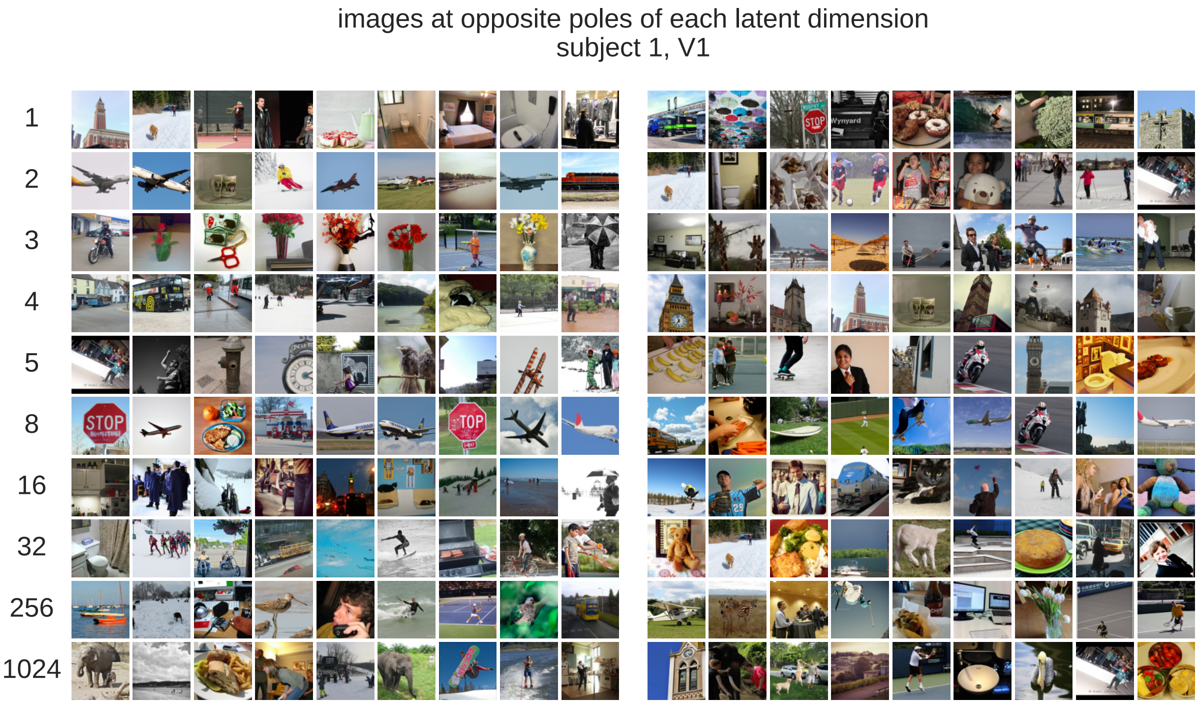

Figure 8: **Visualizing images from the dataset that strongly activate different latent dimensions of V1.** The second latent dimension appears sensitive to airplanes/trains while the third latent dimension is activated strongly by flowers in vases, and the fourth is activated by clocktower-like buildings. However, we expect that these patterns are driven by low-level features such as spatial orientation that covary strongly with these semantic features in these images.

> The authors should also at least speculate on, and ideally show, what information is or may be contained in the higher dimensions that they report here (as these are potentially the most novel observation, to my eye). Could they reflect retinotopic position selectivity? information about exemplar-level differences between objects? Nuances of texture or material properties? The authors critique brain mapping studies as being fundamentally limited in the number of dimensions that can be visualized in brain maps. This is fair enough; however, a description of what features the dimensions capture need not be limited to brain maps per dimension or direct interpretations of each dimension; what space does the neural activity span? what distinctions among stimuli or brain states can be decoded from brain activity with low dimensions only, and with the additional dimensions revealed? To me, the lack of (any) interpretation of the dimensions in this manuscript puts a strong limit on the insights offered by the manuscript.

We thank the reviewer for pushing us to speculate on this topic. We are indeed very interested in developing theories of the representational content at high-rank dimensions, which have largely been overlooked in previous work due to methodological and data limitations. As the reviewer points out, our original submission did not include much discussion of this topic. To address this, we have now added new text to the Discussion section in which we speculate about the nature of high-rank representational dimensions and discuss specific hypotheses for future work (lines 464-475).

Additionally, we would like to emphasize that the focus of the current manuscript is on revealing the full extent and statistical structure of cortical image representations, which had not been fully understood in the previous literature. To accomplish this, we developed a new methodological approach for revealing aspects of cortical representation that conventional methods are insensitive to. We addressed our central question through a series of rigorous cross-validated and cross-subject analyses in multiple brains regions, in two large-scale fMRI datasets, and with direct comparisons to a conventional RSA approach, and we corroborated our findings through many additional analyses and

supplementary figures. Together, our results reveal a fundamental property of human visual cortex representation that had not previously been understood. As noted in response to this reviewer's first major comment, our findings contrast with previous low-dimensional hypotheses in the literature, they have direct implications for theories of information processing along the visual hierarchy, and they have broad implications for how neuroscientists approach the study of visual representations. Finally, though the challenge of interpreting the underlying dimensions of vision remains open, we see this as an exciting future direction, and we believe that our methods open promising new possibilities for tackling this challenge.

> It's not clear that the highest-rank dimensions (beyond 100 dimensions or so) are reliably found across subjects. As far as I can see, the authors only perform significance tests for one pair of subjects (in Fig 3), and in that figure it's not totally clear that the highest-rank bins of dimensions explain reliably more variance than chance. (Perhaps due to the logarithmic Y axis, the error bars span the P cutoff values.) In figure S4, there seems to be a substantial falloff of variance explained beyond ~100 dimensions for many pairs of subjects. Also, the same calculation of chance in Fig 3 should be applied to all the subjects in figure S4 as well, and some summary analysis should be provided to assess reliability of high-rank dimensions across all subjects. What the authors do and do not consider to be reliable should be made very clear. I realize this will be computationally intensive, but this is an important point, because whether or not those higher-rank dimensions are reliable seems to be the difference between finding MORE dimensions than past studies and finding a truly scale-free representation.

We agree with the reviewer's characterization here: we are only able to consistently detect ~100 dimensions that are reliably shared across subjects, primarily due to the limited number of stimuli that are seen by all the subjects (766 images). As a relevant comparison, in the within-subject analyses, there are ~10,000 images seen at least twice, and here we can reliably detect ~1,000 dimensions within each subject. To demonstrate how spectra vary depending on the number of stimuli available, we compute within-individual spectra for an example subject using different numbers of stimuli (Figure 9). This analysis shows that as the number of stimuli decreases, there is a corresponding decrease in the range of significant dimensions and the amplitude of the highest-rank dimensions.

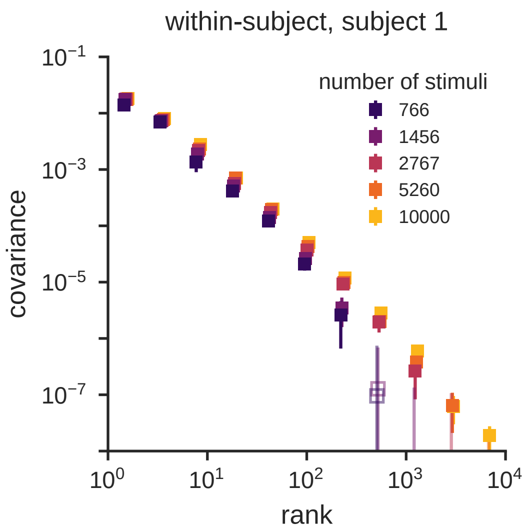

Figure 9: **Varying the number of stimuli used for cross-decomposition.** Within-individual covariance spectra for an example subject (subject 1) are computed using different numbers of stimulus images, ranging from 766 (the number of shared images seen by all participants in the experiments) to 10,000. We note that with fewer stimuli, reliable signal at high-ranks is not detectable. Open symbols denote data that are not significant at $p < 0.001$ (permutation tests, $N = 5000$).

To address the reviewer's concern and provide more evidence that shared visual cortex representations are scale-free, we performed a new set of analyses on another large-scale dataset, THINGS fMRI, in which the same 8,640 natural images of objects were seen by each of 3 subjects. We repeat our between-subject cross-decomposition analysis and find that from V1 to V4 and LOC, there are power-law covariance spectra that span >100 dimensions (in fact, all these spectra approach the limit determined by the size of the ROIs) (Figure 10).

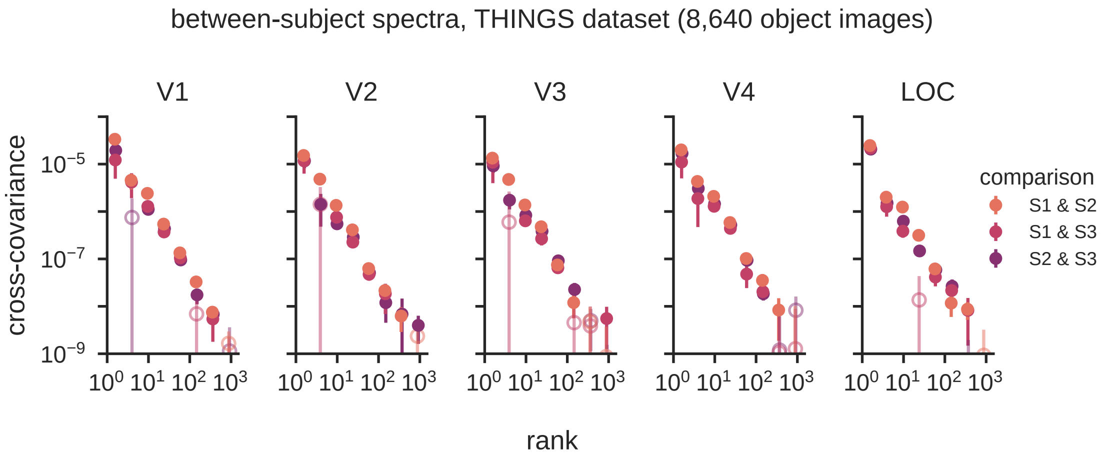

Figure 10: **Power-law spectra of between-subject covariance in the THINGS dataset of fMRI responses to object images.** This plot shows between-subject covariance spectra of visual cortex responses in V1 through V4 and object-selective lateral occipital complex (LOC) from the THINGS-data fMRI dataset (Hebart et al., 2023). All spectra are normalized to account for differences in the number of voxels across participants and averaged within bins of exponentially increasing width and across 8 folds of cross-validation. Error bars denote standard deviations across these 8 folds. Open symbols denote data that are not significant at $p < 0.001$ (permutation tests, $N = 5000$).

As suggested by the reviewer, we have improved all the spectra shown in the manuscript by using open symbols to represent data points that are not significantly above the null distribution. These are typically the same points where large error bars overlap with the X-axis due to the logarithmic scale. We note that we see similar trends across all pairs of participants. We have added an extra panel to Figure S4 showing a summary across participants, where we average all 28 pairwise comparisons of subjects to produce a single spectrum. This averaged spectrum shows that we can detect reliable stimulus-related signal over slightly more than two orders of magnitude in ranks.

> Relatedly, it would help to discuss more how measurement noise affects the estimates of reliability of the highest-rank dimensions.

The analyses presented in Figure 9 show that reliability diminishes for dimensions near the upper limit determined by the number of stimuli. This suggests that the highest rank dimensions are the most susceptible to noise and misestimation. However, interestingly, these analyses also show that the reliability of these same ranks can be dramatically improved by simply increasing the number of stimuli. We have added new text to the Results section (lines 226-229) to describe these points.

> The authors raise the point that the number of dimensions revealed in the brain may depend on the stimulus set; they write: "The scale-free nature of these representations implies that their dimensionality is ill-defined: estimates of effective dimensionality for visual cortex likely reflect the properties of a given dataset such as the number and richness of stimuli or the number of recording channels rather than an intrinsic property of cortical representation." This argument would be more persuasive with a demonstration that other stimulus sets yield fewer dimensions, or a faster falloff of covariance over ranks. A good option for such an analysis would be functional localizer and/or PRF mapping stimuli included for the same participants in NSD. There are fewer unique TRs for these stimulus sets, but demonstrating that the

> elicited responses show a faster falloff of covariance by rank would make clear the degree to which the estimated dimensionality of the visual representation depends on the stimulus set.

To clarify, we did not mean to suggest that the spectrum will be strongly dependent on the details of the types of images used. Rather, we were trying to make a more general point that estimates of effective dimensionality should be interpreted with caution. They do not indicate the actual number of dimensions that make up the representational code of a given brain region (despite some previous work making claims of this sort, e.g., Lehky et al. (2014) and Lehky and Tanaka (2016). Instead, these estimates are more likely to reflect the size of the dataset (e.g., the number of stimuli or the number of recording channels). To avoid confusion, we have now edited the text so that it no longer mentions stimulus richness and instead focuses on dataset size.

We also want to note here that previous work in mouse visual cortex shows that the neural spectrum is remarkably robust to manipulations of image properties (Stringer et al., 2019). Multiple distinct stimulus sets yielded the same power-law spectrum in mouse V1, and only highly artificial and extremely low-dimensional stimuli (e.g., images reduced to just 4-8 dimensions) yielded a more rapidly decaying spectrum. The stimuli in the fMRI localizer and in the NSD synthetic dataset are complex enough that we would expect them to elicit the same power-law spectrum. In sum, we do not expect that the shape of the neural spectrum will be highly sensitive to image properties (so long as the stimuli are not extremely impoverished). To put this another way, while the details of the stimuli will certainly be reflected in patterns of activity distributed over a high-dimensional spectrum, we do not expect that they will alter the global shape of the spectrum.

> Broadly speaking, it would be useful to describe in more intuitive terms what the dimensions they report are. As far as I understand, each dimension derived from the cross-decomposition is a pattern of responses across (visual) voxels for a particular subject. Similarly high values in different voxels for each dimension reflect commonalities in what visual factor or factors drives the voxels. Is this approximately correct? Some language to this effect (obviously corrected if I have misapprehended anything) would be helpful to the reader.

The reviewer's description is correct and as suggested, we have added a more intuitive description of these dimensions to the text (lines 112-114). Each dimension derived from cross-decomposition is analogous to an eigenvector from PCA, corresponding to a linear combination of voxels in a particular subject's visual cortex. For each dimension, the pattern of weights over voxels describes the contribution of each voxel to that dimension.

2.2 Minor comments

> In all cross-subject analyses, each dimension is found by decomposing the covariance matrix of a pair of subjects, and evaluated on held-out data between those same two subjects. Thus, there does not seem to be any guarantee that the dimensions are consistent beyond that pair. This, to me, means that the word 'universal' should be used with care. Referring to a "universal scale-free spectrum" seems fair, but a "universal scale-free code" seems to imply that a universal code or encoding has been found, which to me strongly implies that the same set of dimensions has been found for all subjects, which is not the case. So the phrase "universal scale-free code" seems to be overreaching the data.

We agree with the reviewer and have removed the specific phrasing "universal scale-free code". However, we would like to note here that in our pairwise analyses, all pairs of subjects share a high-dimensional subspace limited only by the number of stimuli/voxels, which strongly suggests that all subjects share an underlying high-dimensional visual representation.

> In figure 3, it would be better to plot the mean of the null distribution rather than or in addition to the few examples of permuted distributions.

As suggested, we have plotted the mean of the null distribution in addition to the samples of the permuted spectra. As expected, the empirical mean of this null distribution is zero at all rank bins.

> The graphs of spectral covariances and spectral correlations do not show values at every possible rank, but only at a sampling of ranks (at the centers (?) of bins of ranks). The authors write in the caption of Figure 2 that the covariances are "averaged within bins of exponentially increasing width", but this is the only information I could find on the bins. The authors should specify precisely what the bins are in the methods (and if these differ across subjects due to different numbers of stimuli presented and/or voxels, this should be clarified), and how the specific logarithmic spacing of bins were chosen.

We thank the reviewer for pointing out the omission and have added these details to the Methods (lines 541-548). We used the same bins across all spectra depicted in the manuscript. These logarithmically spaced bins were constructed by geometrically sampling the maximum range of possible ranks [1, 10,000] with an approximate density of 3 bins per decade, corresponding to 11 total bins across these 4 orders of magnitude. This density was chosen as a tradeoff between maximizing the spectral resolution by increasing the number of bins and maximizing the signal-to-noise ratio in high-rank bins by averaging over a larger number of ranks. Our results are qualitatively similar when using different numbers of bins (Figure 13).

> It would be useful for the authors to give more intuition for how to think about the covariance values in many of their plots. The authors note that the values are not strictly positive, as within-set variance explained by PCs would be, but are they bounded in any way? Does the normalization applied to the covariance estimates make the estimated covariances cumulatively sum to 1 (or approximately 1)?

As suggested by the reviewer, we have now added text to explain this in more detail in the Methods section (lines 550-555). Specifically, since the responses of all voxels are independently z-scored using their means and standard deviations across training images, each voxel has approximately unit variance across the entire dataset, making the total variance of the dataset approximately equal to the number of voxels. Our normalization procedure ensures that the covariance spectra sum to 1 on the training set, and would sum to 1 on the test set if all the stimulus-related variance was reliable in the generalization test.

> In the introduction, the authors should cite a reference for their estimate of the number of neurons in human visual cortex. "the effective dimensionality may not have an intrinsic upper bound other than the total number of neurons, which is about 109 in human visual cortex."

We thank the reviewer for pointing out the omission. We have added the relevant citation (line 96).

> In the methods and the results, the authors state that approximately 1,000 images were shown to all subjects, and note that some subjects didn't see all images. The authors should be more precise. 907 images were shown to two participants; what was done about the other 93 images that these participants didn't see in the cross-subject decompositions? It seems that n (number of stimuli) has to be consistent for the math to work, so: Were only the common 907 images used in the cross-subject decompositions? were different numbers of images used for cross-decomposition in different pairs of subjects, each depending on the minimum number of common images between each pair of subjects? (I think so, based on what the authors have written, but this could be clearer.)

We thank the reviewer for pointing out this potential source of confusion. We have updated the Methods to clarify exactly which stimuli were used for the analyses (lines 497-501). As a summary, we used the subset of 766 images that were seen at least twice by all participants for all between-subject analyses, even if some pairs of subjects may have more images in common. This ensures that comparisons between all pairs of subjects are fair and rely on the same underlying stimulus set, with the tradeoff that we do not use all the available data and thereby reduce signal-to-noise.

> on p 13, the authors write "However, as we have shown, human brain representations are expressed using the full dimensionality of the available space (ultimately defined by the number of neurons)" This seems to be reaching beyond the data; better to say "ultimately defined by the number of voxels" (or measurements made)

We thank the reviewer for the correction: we indeed meant to say "voxels" and not "neurons".

3 Reviewer #3

In this study, the authors analyze stimulus-related fMRI responses from the Natural Scenes Dataset (NSD) using spectral analysis of cross-validated cross-covariance. The main findings are that these responses exhibit high dimensionality and follow a scale-free (power-law) spectrum across four orders of magnitude. Furthermore, the authors show that many of these dimensions are shared across NSD participants, spanning approximately three orders of magnitude. This work complements previous findings in mouse V1 by Stringer et al. (2019, Nature), who similarly observed scale-free population activity patterns.

This is an interesting and timely study that is likely to attract considerable attention within the neuroscience community—and potentially spark debate. The paper is well written, clear, and engaging. The overall approach, which uses cross-validated spectral analysis of stimulus-related responses, is both straightforward and well motivated. However, given that the central claims regarding scale-free, high-dimensional representations rest entirely on the validity of the analyses (as the work is observational in nature), I believe that additional scrutiny prior to publication is warranted. Below, I outline several issues the authors may wish to address before the paper is published.

Thank you!

1. Given the spatial tuning properties of fMRI voxels, a certain degree of high dimensionality is expected. It would be beneficial to compare the observed dimensionality to that predicted by a linearized Gabor wavelet encoding model. How much of the observed dimensionality exceeds what would be expected from spatial tuning alone? Could the observed spectrum be fully explained by such a model?

The reviewer is correct that spatial tuning alone would lead to high-dimensional representations, especially in V1 where receptive fields are small. In previous work, Stringer et al. (2019) demonstrated that a Gabor receptive field model itself has a power-law covariance spectrum, though with an exponent that differs significantly from that of mouse V1 responses to natural images, suggesting that neural V1 representations are not fully captured by a Gabor model. While visual features extracted using a Gabor filter bank explain a significant amount of variance in primary visual cortex (V1), they perform less well in explaining higher-level visual regions. The power-law covariance spectra we report here are found beyond V1 in both mid- and high-level visual cortex where voxels have much larger receptive fields, and where previous work has suggested that representations are low-dimensional (e.g., Fusi et al. (2016), Lehky et al. (2014), Lehky and Tanaka (2016)). Together, this suggests that a Gabor model may not fully explain the observed spectra in all these regions. However, we agree with the reviewer that comparing neural responses to a Gabor model using cross-decomposition provides a valuable baseline, and we have now done so in the revised manuscript.

Specifically, we generated a Gabor filter bank using quadrature pairs of Gabor filters, each of which obeys Equation 1:

$$g(x, y; \sigma, \gamma, f, \theta, \psi) = \exp\left(-\frac{x'^2 + \gamma^2 y'^2}{2\sigma^2}\right) \cos\left(2\pi f x' + \psi\right) \tag{1}$$

where x and y denote the location of the center of the filter relative to the image; $x' = x\cos\theta + y\sin\theta$ and $y' = -x\sin\theta + y\cos\theta$; σ and γ denote the scale and aspect ratio of the Gaussian envelope; and f, θ and ψ denote the spatial frequency, orientation, and phase of the sinusoid.

Following Cadena et al. (2019), the feature space of this Gabor filter bank is the concatenation of three sets of features: (i) simple cell responses from each "odd" filter (phase $\psi = 0$), (ii) simple cell responses from each "even" filter (phase $\psi = \pi/2$), and (iii) complex cell responses modelled as the sum of the squares of (i) and (ii), which captures the "energy" of each quadrature pair. We use the following parameters: 3 scales ($\sigma = 0.025, 0.050, 0.075$, where the square image has relative side length 1); 1 aspect ratio ($\gamma = 1$); 3 frequencies at each scale σ ($f = 0.25\sigma, 0.5\sigma, 0.75\sigma$); 8 orientations θ uniformly spaced from $[0, \pi]$; and $8 \times 8 = 64$ spatial locations (x, y with 8 uniformly spaced locations in $[-0.45, 0.45]$, where the image center is at $(0, 0)$). This produces 4,608 quadrature pairs, leading to a total of 13,824 features in the feature space. As a preprocessing step, each of these features is z-scored across all 73,000 stimuli in the Natural Scenes Dataset.

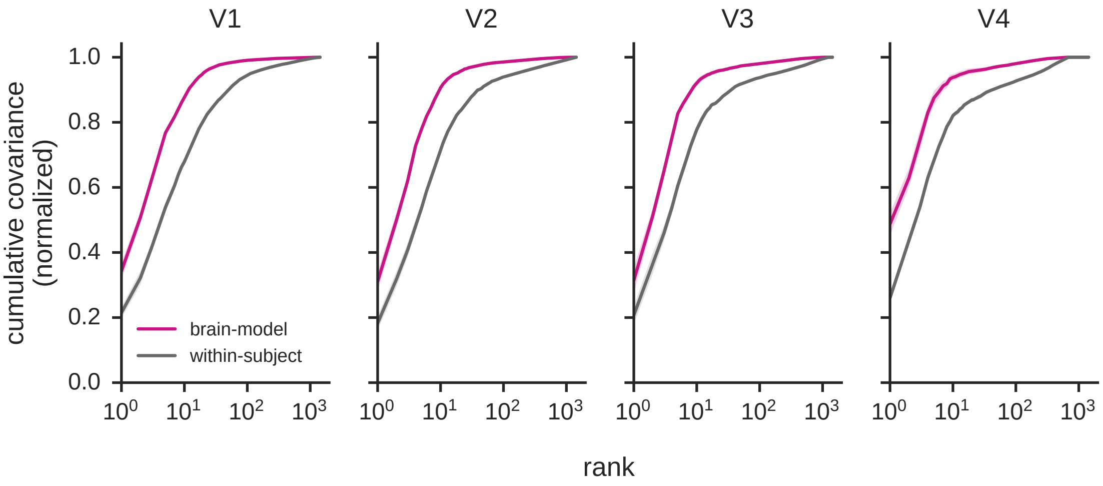

Figure 11: **A Gabor filter bank cannot fully explain the observed high-dimensional structure of the neural population responses.** (Pink) The normalized cumulative shared variance between (i) neural responses on the first stimulus presentation and (ii) neural responses on the second stimulus presentation predicted by the Gabor model. (Gray) The normalized cumulative shared variance between the neural responses to the first and second presentations of the stimuli (within-subject spectrum shown in the main manuscript). Data are shown for example subject 1. In all regions from V1 through V4, the Gabor model is consistently lower-dimensional than the neural data, as demonstrated by the more rapid increase in cumulative variance.

We use L_2-regularized linear regression to fit the response patterns of each voxel in an example subject (subject 1) using features from the Gabor filter bank as predictors. The data were split into two halves containing responses to 5,000 stimuli each, used as the training and test sets respectively. The optimal shrinkage parameter was estimated for each voxel using leave-one-out cross-validation on the training set (out of 30 values logarithmically sampled from 10^4 to 10^8). Then, we used cross-decomposition to compute the spectrum of reliable stimulus-related variance shared between (i) neural responses on the first presentation of the stimuli and (ii) neural responses on the second presentation of the stimuli predicted by the Gabor model (Figure 11, pink). We compare these to the within-subject covariance spectrum reported in the manuscript, which compares actual neural responses on the first and second presentations of the stimuli (Figure 11, gray). To highlight differences between these spectra, we plot the *cumulative* variance of these shared latent dimensions (as in Figure 2F of Stringer et al. (2019)). Replicating Stringer et al. (2019), we find that the Gabor model is consistently lower-dimensional than the neural data in all regions from V1 through V4, as demonstrated by the more rapid increase in cumulative variance. This suggests that the observed dimensionality of the neural data cannot be fully explained by spatial tuning alone. We have added these results to the revised manuscript (Figure S4 and lines 288-290 in the Results).

2. The authors briefly acknowledge the assumption of linearity in their discussion but do not provide concrete controls for how violations of this assumption might affect their conclusions. Two plausible scenarios are not ruled out: (1) stimulus-related neural responses may lie in a low-dimensional nonlinear manifold, which PCA-based analyses would not capture (this possibility is mentioned in the discussion); and (2) stimulus-related neural responses may lie in a low-dimensional linear subspace that is distorted by nonlinearities in the hemodynamic response. In both cases, the assumption of linearity could lead to an overestimation of dimensionality. These concerns could be addressed through simulations quantifying their potential impact, through nonlinear analysis methods, and/or by comparison with spiking data.

First, we want to clarify that there is an important distinction between linear and nonlinear dimensionality, and we caution against conflating these concepts. Linear and nonlinear methods measure fundamentally different quantities that have different interpretations, and there is no single meaning of "dimensionality" that applies to both.

It is an open question whether there may be a way of understanding vision as operating on some nonlinear manifold whose intrinsic dimensionality is lower than the linear dimensionality of neural population activity. However, this is a distinct question from the one addressed by our work, which focuses on understanding how stimulus-related variance is distributed across linear dimensions. Linear dimensions of neural population activity are important to understand in their own right, and they have long been the primary focus of representational modeling efforts in both systems and cognitive neuroscience. For example:

- The vast majority of representational modeling studies in the field use linear methods (e.g., linear regression, linear classifiers, RSA with linear distance metrics).
- Highly relevant previous studies of visual population codes have focused on linear latent dimensions (Lehky et al., 2014; Stringer et al., 2019).
- A prevailing theory argues that the visual system makes sensory information accessible by linearizing it in neural population activity such that it can be read out be a downstream linear decoder (i.e., “untangling”) (DiCarlo & Cox, 2007)

Regarding the reviewer’s second point, we agree that the hemodynamic response could potentially yield a distorted view of the actual underlying spectrum in *neural* activity. However, as shown below (Figure 12), we find highly consistent results when we apply our cross-decomposition procedure to calcium imaging measurements of single neuron responses in mouse V1 (using data from Stringer et al. (2019)). Thus, our method reveals a similar result when applied to direct measurements of neural activity.

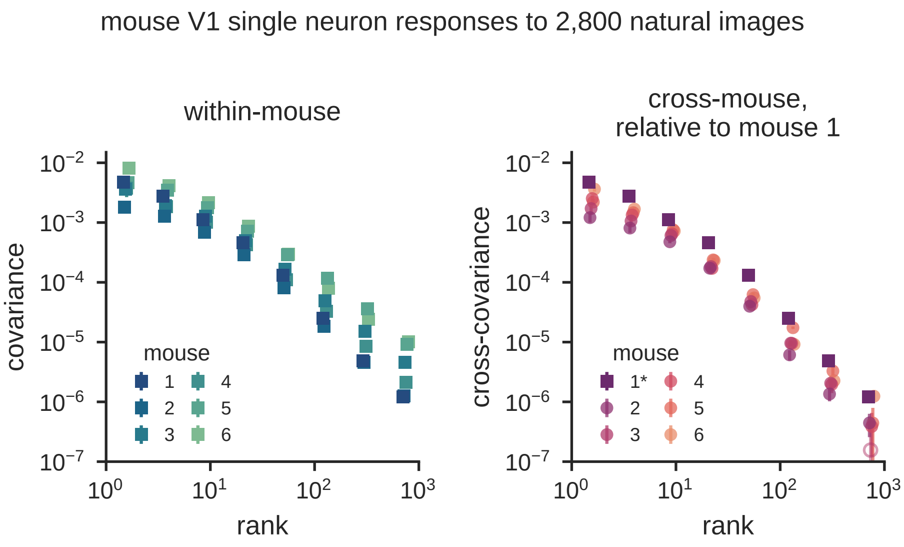

Figure 12: **Within- and between-individual power-law covariance spectra in mouse V1 single neuron responses to natural images.** Within-mouse (left) and between-mouse (right) covariance spectra computed for mice measuring the spectrum of reliable variance in neural responses. All spectra are normalized to account for differences in the number of neurons across animals and averaged within bins of exponentially increasing width and across 8 folds of cross-validation. Error bars denote standard deviations across these 8 folds. Open symbols denote data that are not significant at $p < 0.001$ (permutation tests, $N = 5000$).

3. While binning the spectrum improves statistical power and aids visualization, it also makes it more difficult to visually assess whether the spectrum truly follows a power-law (i.e., appears as a straight line in log-log coordinates). To strengthen the claim of scale-free structure, it would be helpful to conduct statistical model comparisons between a power-law fit and plausible alternatives. These comparisons should ideally be based on the unbinned spectral coefficients to avoid smoothing out potential deviations.

The assumptions behind the statistical tests developed to evaluate whether a distribution empirically obeys a power-law (Clauset et al., 2009) do not apply in our case, since our spectra are (i) cross-validated, making them potentially negative for a subset of ranks and (ii) binned to extract signal in noisy regimes, thus reducing the number of samples. With such a statistical estimator, permutation tests are the gold standard because they rely on minimal assumptions on the data-generating process (e.g., models of the noise distribution required for a maximum likelihood estimator). For these reasons, we used permutation tests as the primary tool for statistical inference in our work, with a focus on determining the range of dimensions over which significant signal is detected. We also want to note that binning—which is a common method in fields that regularly deal with power-law distributed data—does not change the fundamental shape of the spectrum. We confirmed this in our own analyses by replotting the data for an example subject (subject 1) at various levels of binning (Figure 13). As expected, when there are more bins per decade, the spectra are noisier, especially in the tail. However, at all binning densities, the power-law shape of the spectrum is preserved.

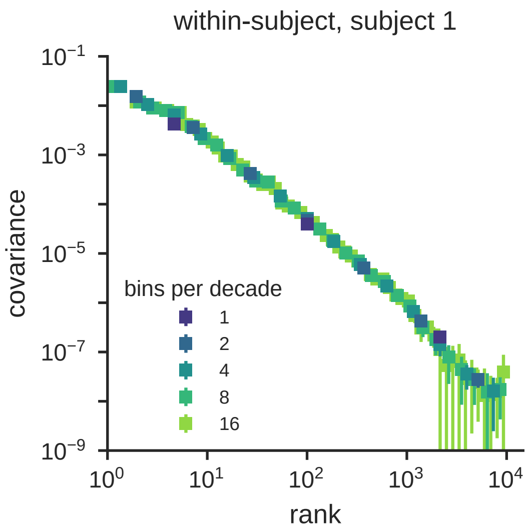

Figure 13: **Varying the number of bins used to visualize spectra.** Within-individual covariance spectra for an example subject (subject 1) are visualized at various levels of binning, ranging from 1 bin per decade to 16 bins per decade (all other spectra reported in the paper are visualized with 3 bins per decade). The shape of the power-law spectrum is preserved at all levels of binning. Error bars denote standard deviations across 8 cross-validation folds.

4. The NSD synthetic dataset has been very recently released. Performing measurements on synthetic stimuli could serve as an important test case for the generality of the authors' claims, similarly to the usage of synthetic stimuli in Stringer et al., 2019.

We are interested in the question of how cortical spectra might be affected, if at all, by analyzing out-of-distribution stimuli. However, we prefer to leave this as a future direction for several reasons. First, while the NSD-synthetic dataset seems very promising (Gifford et al., 2025), we would prefer to wait until the paper for this dataset is peer-reviewed and published before we start analyzing and publishing on these data ourselves. Second, our manuscript already contains many cross-validated and cross-subject analyses in multiple brains regions, in two large-scale fMRI datasets (see below), and with many additional analyses and supplementary figures that corroborate our findings. Third, in a new set of analyses that are now reported in the revised manuscript, we have strengthened the generality of our claims by reproducing our key results using the THINGS-data fMRI dataset (Hebart et al., 2023) (Figure 14).

The THINGS dataset contains data from 3 participants who viewed 8,640 images of objects centered on a naturalistic background, with 12 images from each of 720 object categories ("concepts"). This number of unique stimuli is comparable in scale to the Natural Scenes Dataset (~10,000 images per participant) (Allen et al., 2021). However, in the THINGS fMRI dataset, each image is seen only once by each participant, making it impossible to run our within-subject cross-decomposition analysis, which relies on having repeated measurements of cortical responses to

the same stimuli. However, the same 8,640 images are seen by all three participants in the dataset, allowing us to run our between-subject cross-decomposition analysis with higher statistical power than in the NSD, where only 766 images are shared across participants. As the between-subject analyses are a more stringent test than the within-subject analyses, this makes for a compelling replication of our results in the NSD dataset.

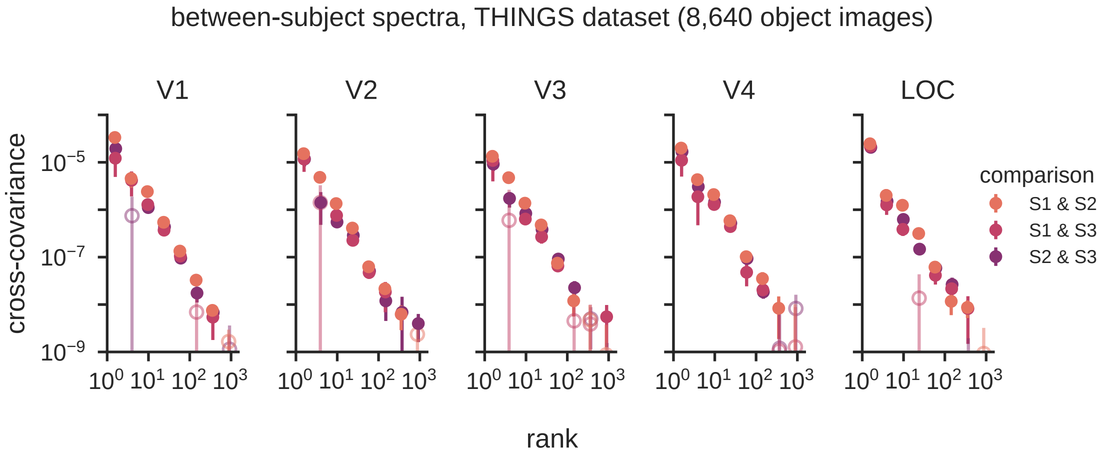

Figure 14: **Power-law spectra of between-subject covariance in the THINGS dataset of fMRI responses to object images.** This plot shows between-subject covariance spectra of visual cortex responses in V1 through V4 and object-selective lateral occipital complex (LOC) from the THINGS-data fMRI dataset (Hebart et al., 2023). All spectra are normalized to account for differences in the number of voxels across participants and averaged within bins of exponentially increasing width and across 8 folds of cross-validation. Error bars denote standard deviations across these 8 folds. Open symbols denote data that are not significant at $p < 0.001$ (permutation tests, $N = 5000$).

5. It is somewhat unclear which methodological components are novel and which are adapted from previous work. Clarifying this distinction is important not only for assigning appropriate credit but also for identifying which aspects of the analysis may require further validation, for example through simulation studies. In particular, it would be helpful to clarify whether the correlation coefficient defined in Equation 1, used to quantify representational similarity across latent dimensions, is based on prior work or whether it is introduced here for the first time.

Although our approach introduces a new perspective on the analysis of neural data, it is based on well-established statistical methods. Thus, the statistical estimators in our work are not new—they are just unconventional in the field of neuroscience. Below are specific comments on our key statistical methods:

- The cross-decomposition procedure is well established. It goes by several names in the literature, including Procrustes transformation and PLS-SVD. In the neuroscience literature, the Procrustes transform has been used for hyperalignment. In the Results section, we explain the connection to hyperalignment and credit Haxby et al. (2011).
- Characterizing how variance decays across ranks is standard in PCA.
- Logarithmic binning is standard in fields that regularly analyze power-law distributed data (Lin & Newberry, 2023; Milojević, 2010).
- The specific cross-correlation coefficient in Equation 1 has not been reported before in the neuroscience literature, but it is akin to noise-ceiling normalization, which is now a common method in cognitive and systems neuroscience. More broadly, it follows the same formula as a more general class of correlation estimators that normalize the cross-covariance between two measurements X and Y by the geometric mean of their individual variances (e.g., the Pearson correlation coefficient).

6. The treatment of RSA and related metrics is interesting, but it may benefit from a broader consideration of tradeoffs in representational comparison methods. While it is true that RSA, like other variance-weighted metrics, is largely insensitive to high-rank (i.e., low-variance) components, alternative approaches that do not apply variance weighting—such as canonical correlation analysis—have been shown to suffer from poor specificity (Kornblith et al., 2019 ICML). It may be useful to connect this work more explicitly to that literature and to consider the broader tradeoff between sensitivity to weak but potentially meaningful components and the risk of detecting spurious alignments.

We agree with the reviewer and have added this point to the Discussion section (lines 424-427). We emphasize that our cross-validation procedure guards against detecting spurious alignments.

References

Allen, E. J., St-Yves, G., Wu, Y., Breedlove, J. L., Prince, J. S., Dowdle, L. T., Nau, M., Caron, B., Pestilli, F., Charest, I., Hutchinson, J. B., Naselaris, T., & Kay, K. (2021). A massive 7t fMRI dataset to bridge cognitive neuroscience and artificial intelligence. *Nature Neuroscience*, *25*(1), 116–126. https://doi.org/10.1038/s41593-021-00962-x

Cadena, S. A., Denfield, G. H., Walker, E. Y., Gatys, L. A., Tolias, A. S., Bethge, M., & Ecker, A. S. (2019). Deep convolutional models improve predictions of macaque v1 responses to natural images (W. Einhäuser, Ed.). *PLOS Computational Biology*, *15*(4), e1006897. https://doi.org/10.1371/journal.pcbi.1006897

Clauset, A., Shalizi, C. R., & Newman, M. E. J. (2009). Power-law distributions in empirical data. *SIAM Review*, *51*(4), 661–703. https://doi.org/10.1137/070710111

DiCarlo, J. J., & Cox, D. D. (2007). Untangling invariant object recognition. *Trends in Cognitive Sciences*, *11*(8), 333–341. https://doi.org/10.1016/j.tics.2007.06.010

Fusi, S., Miller, E. K., & Rigotti, M. (2016). Why neurons mix: High dimensionality for higher cognition. *Current Opinion in Neurobiology*, *37*, 66–74. https://doi.org/10.1016/j.conb.2016.01.010

Gifford, A. T., Cichy, R. M., Naselaris, T., & Kay, K. (2025). A 7t fmri dataset of synthetic images for out-of-distribution modeling of vision. https://doi.org/10.48550/ARXIV.2503.06286

Haxby, J. V., Guntupalli, J. S., Connolly, A. C., Halchenko, Y. O., Conroy, B. R., Gobbini, M. I., Hanke, M., & Ramadge, P. J. (2011). A common, high-dimensional model of the representational space in human ventral temporal cortex. *Neuron*, *72*(2), 404–416. https://doi.org/10.1016/j.neuron.2011.08.026

Hebart, M. N., Contier, O., Teichmann, L., Rockter, A. H., Zheng, C. Y., Kidder, A., Corriveau, A., Vaziri-Pashkam, M., & Baker, C. I. (2023). Things-data, a multimodal collection of large-scale datasets for investigating object representations in human brain and behavior. *eLife*, *12*. https://doi.org/10.7554/elife.82580

Huth, A. G., Nishimoto, S., Vu, A. T., & Gallant, J. L. (2012). A continuous semantic space describes the representation of thousands of object and action categories across the human brain. *Neuron*, *76*(6), 1210–1224. https://doi.org/10.1016/j.neuron.2012.10.014

Khosla, M., Ratan Murty, N. A., & Kanwisher, N. (2022). A highly selective response to food in human visual cortex revealed by hypothesis-free voxel decomposition. *Current Biology*, *32*(19), 4159–4171.e9. https://doi.org/10.1016/j.cub.2022.08.009

Lehky, S. R., Kiani, R., Esteky, H., & Tanaka, K. (2014). Dimensionality of object representations in monkey inferotemporal cortex. *Neural Computation*, *26*(10), 2135–2162. https://doi.org/10.1162/neco_a_00648

Lehky, S. R., & Tanaka, K. (2016). Neural representation for object recognition in inferotemporal cortex. *Current Opinion in Neurobiology*, *37*, 23–35. https://doi.org/10.1016/j.conb.2015.12.001

Lin, Q., & Newberry, M. (2023). Seeing through noise in power laws. *Journal of The Royal Society Interface*, *20*(205). https://doi.org/10.1098/rsif.2023.0310

Milojević, S. (2010). Power law distributions in information science: Making the case for logarithmic binning. *Journal of the American Society for Information Science and Technology*, *61*(12), 2417–2425. https://doi.org/10.1002/asi.21426

Portilla, J. (2000). *International Journal of Computer Vision*, *40*(1), 49–70. https://doi.org/10.1023/a:1026553619983

Posani, L., Wang, S., Muscinelli, S., Paninski, L., & Fusi, S. (2024). Rarely categorical, always high-dimensional: How the neural code changes along the cortical hierarchy. https://doi.org/10.1101/2024.11.15.623878

Rigotti, M., Barak, O., Warden, M. R., Wang, X.-J., Daw, N. D., Miller, E. K., & Fusi, S. (2013). The importance of mixed selectivity in complex cognitive tasks. *Nature*, *497*(7451), 585–590. https://doi.org/10.1038/nature12160

Shirakawa, K., Nagano, Y., Tanaka, M., Aoki, S. C., Majima, K., Muraki, Y., & Kamitani, Y. (2024). Spurious reconstruction from brain activity. https://doi.org/10.48550/ARXIV.2405.10078

Sorscher, B., Ganguli, S., & Sompolinsky, H. (2022). Neural representational geometry underlies few-shot concept learning. *Proceedings of the National Academy of Sciences*, *119*(43). https://doi.org/10.1073/pnas.2200800119

Stringer, C., Pachitariu, M., Steinmetz, N., Carandini, M., & Harris, K. D. (2019). High-dimensional geometry of population responses in visual cortex. *Nature*, *571*(7765), 361–365. https://doi.org/10.1038/s41586-019-1346-5

Tarhan, L., & Konkle, T. (2020). Sociality and interaction envelope organize visual action representations. *Nature Communications*, *11*(1). https://doi.org/10.1038/s41467-020-16846-w

Yan, Y., Goodman, J. M., Moore, D. D., Solla, S. A., & Bensmaia, S. J. (2020). Unexpected complexity of everyday manual behaviors. *Nature Communications*, *11*(1). https://doi.org/10.1038/s41467-020-17404-0
