# Supplementary material for: Universal scale-free representations in human visual cortex
Source: PLoS Comput Biol. 2025 Nov 21;21(11):e1013714. doi: 10.1371/journal.pcbi.1013714 (PMC12654933; doi:10.1371/journal.pcbi.1013714)
Supplement: Attachment — Submitted filename: response.pdf [file pcbi.1013714.s016.pdf]

Response to reviewer comments — Round 2

Raj Magesh Gauthaman[1], **Brice Ménard**[2,1,3], **Michael F. Bonner**[1]
[1]Department of Cognitive Science, [2]Department of Physics and Astronomy
Johns Hopkins University, Baltimore, MD
[3]Santa Fe Institute, Santa Fe, NM
{rgautha1, bmenard1, mfbonner}@jh.edu

1 Reviewer #1

> I thank the authors for their efforts in addressing my concerns. Their revisions have substantially clarified the paper, and I find the core claims more compelling.

We thank the reviewer for this positive assessment.

> However, I remain concerned that the calcium imaging analyses suffer from the same issues related to spatial smoothing as fMRI. To convincingly rule out these concerns, it would have been preferable to include a neurophysiology dataset less susceptible to spatial smoothing artifacts (such as the recently released NSD dataset from the Roelfsema Lab).

The calcium imaging data we analyze (Stringer et al., 2019) uses the `suite2p` toolbox (Pachitariu et al., 2016) to preprocess the two-photon microscopy data and the non-negative deconvolution procedure used by `suite2p` for spike extraction has been shown to robustly detect spikes in datasets with ground-truth electrophysiology (Pachitariu et al., 2018). As a result, we expect that there are minimal spatial smoothing artifacts in these mouse V1 data, especially relative to the extreme spatial smoothing inherent to fMRI data.

However, we agree with the reviewer that replicating the results in a monkey neurophysiology dataset would be even stronger, and we have taken their suggestion to analyze the recently published THINGS ventral stream spiking dataset (TVSD) from the Roelfsema lab (Papale et al., 2025). Here, multi-unit activity (MUA) from 1,024 microelectrodes (implanted in V1, V4, and IT) was recorded from two macaques in response to over 25,000 natural images. We perform our between-subject cross-decomposition analysis using the pre-processed MUA data to measure the spectrum of stimulus-related variance that is reliably shared between these two macaques. This analysis revealed a power-law spectrum in the monkey neurophysiology data, thus replicating the findings obtained from human fMRI and mouse calcium imaging. These results are now reported in Figure S12 in the revised manuscript, and they are also reproduced below (Response Figure 1).

2 Reviewer #2

> The authors have done a good job with the revision. The expanded discussion clarifies the relevance of the findings to the field, and they have added important methodological details (regarding number of images in the cross-covariance analyses and the definition of the bins) and statistical tests for the relevant plots. The analysis of how the reliability of the within-subject spectrum depends on the number of stimuli is a nice touch. The dimensionality analysis of the predictions of a fit Gabor model is also very nice.

We thank the reviewer for their comments.

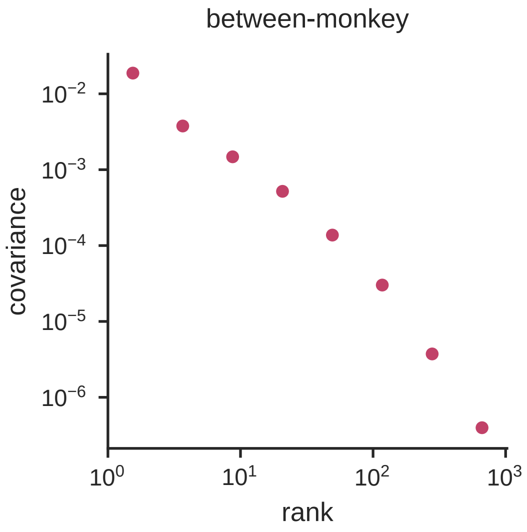

Response Figure 1: **Power-law spectrum of between-monkey covariance in the THINGS ventral stream spiking dataset.** This plot shows the between-monkey covariance spectrum of visual cortex responses in V1, V4, and IT from the THINGS ventral stream spiking dataset (Papale et al., 2025). The spectrum is averaged within bins of exponentially increasing width and across 8 folds of cross-validation. Error bars denote standard deviations across these 8 folds. Open symbols denote data that are not significant at $p < 0.001$ (permutation tests, $N = 5000$). Note that the error bars are small and not visible. This is because this dataset contains twenty times more stimuli than channels, and thus, the covariance estimates are highly consistent across splits of the data.

> The authors have addressed most of the points I raised, but I have a small number of remaining questions and concerns which I am confident the authors can address.
>
> 1. Gabor dimensions & shared dimensions
>
> As noted above, the demonstration that the predictions of a fit Gabor model are lower dimensional than the brain responses themselves is nice. However, I would be very curious to see whether the shared dimensions across participants are also higher dimensional than the Gabor model. It seems straightforward to make a similar plot for cumulative between-subjects covariance, or to add a line for between-subjects covariance to the same plot (fig S4). This would provide a useful test of whether the cross-subjects variance reported here is effectively subsumed by the Gabor model, and the rest of the within-subject variance is idiosyncratic to individuals. I don't view this as strictly necessary, but I think it is a relatively easy analysis to include and would strengthen the paper.

We have run the between-subject analysis suggested by the reviewer and added a second row of panels to Figure S4, analogous to the within-subject analysis in the upper row. This figure is reproduced below (Response Figure 2). The results are highly similar to those from the within-subject analyses.

> 2. Interpretation of dimensions / relation to reported dimensions
>
> The authors have provided visualizations of images at the ends of several dimensions they have uncovered. This is a partial answer to my question, but a more complete answer would relate the dimensions more directly to the dimensions reported in Khosla et al (2022) and Contier, Baker, & Hebart (2024). At least some of the dimensions appear to be similar.

Khosla et al. (2022) report 5 latent components obtained through nonnegative matrix factorization (NMF) of the fMRI responses from the Natural Scenes Dataset (NSD) that are consistent across individuals. These latent components

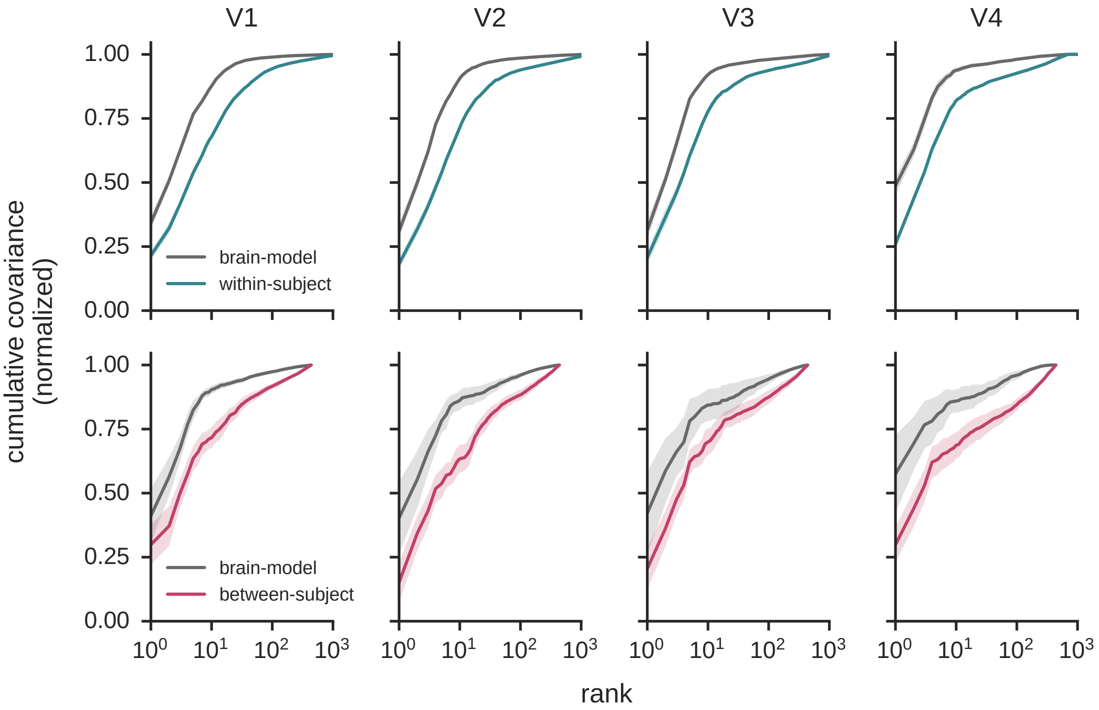

Response Figure 2: **A Gabor filter bank cannot fully explain the observed high-dimensional structure of the neural population responses.** (Top) (Gray) The normalized cumulative shared variance between (i) neural responses on the first stimulus presentation and (ii) neural responses on the second stimulus presentation predicted by the Gabor model. (Green) The normalized cumulative shared variance between the neural responses to the first and second presentations of the stimuli (within-subject spectrum shown in the main manuscript). Data are shown for example subject 1. (Bottom) (Gray) The normalized cumulative shared variance between (i) neural responses for the first subject and (ii) neural responses for the second subject predicted by the Gabor model. (Red) The normalized cumulative shared variance between the neural responses of the first and second subjects (between-subject spectrum shown in the main manuscript). In all regions from V1 through V4, the Gabor model is consistently lower-dimensional than the neural data, as demonstrated by the more rapid increase in cumulative variance. Error bands denote standard deviations across 8 folds of cross-validation. Note that the between-subject analysis (bottom) uses 10 times fewer stimuli than the within-subject analysis (top) and thus has larger error bands.

correspond to faces, bodies, food, text, and scenes and these semantic labels were validated based on human salience ratings from a separate experiment. However, these ratings are not publicly available, precluding their use in a direct comparison with the orthogonal dimensions we obtain from cross-decomposition.

However, we performed an analysis to examine whether the concepts associated with the Khosla components are encoded along individual dimensions from our analyses. We relied on object annotations available in the COCO dataset (Lin et al., 2014) from which the NSD images were sampled. As the object annotations do not include separate "face" and "body" categories, we instead identify images containing objects belonging to the supercategory "person". To probe the "food" component, we identify images containing objects belonging to the supercategories "food" and "food-stuff". To probe the "text" component, we identify images that contain text according to the COCO-Text V2.0 dataset (Veit et al., 2016). Because there is no category label that clearly indicates "scene", and because the Khosla scene ratings are not available, we omit analysis of this component.

To determine whether the latent dimensions we obtain from cross-decomposition contain information about these three stimulus categories (i.e., "person", "food", and "text"), we evaluate whether each dimension separates images with vs. without the category. We focus on the first 10 ranks of dimensions from an example subject (subject 1) and project images with and without the category onto each dimension. We then evaluate whether the distributions of these scores differ significantly for images with vs. without the category (Mann-Whitney U tests). We find that each of these concepts are significantly classified by multiple low-rank dimensions from our analyses, but they do not correspond to specific individual dimensions. These results are now reported in Figure S13 and are reproduced below (Response Figure 3). This figure replaces Figure S11 (which examined "animacy") from our previous submission.

On the reviewer's suggestion, we performed an additional analysis to compare the 66 behavioral dimensions reported in Hebart et al. (2023) and Contier et al. (2024) to the orthogonal latent dimensions obtained from our cross-decomposition analysis of the THINGS fMRI dataset (Hebart et al., 2023). We perform between-subject cross-decomposition of the neural responses in the lateral occipital complex (LOC) from subjects 1 and 2 and project all the images onto the left singular vectors. Then, we average these projections across all images within each of the 720 object categories and correlate each of these neural dimensions with each of the 66 behavioral dimensions (Response Figure 4). Again, as with the dimensions reported by Khosla et al. (2022), we find that there is no clear one-to-one relationship between the latent dimensions from cross-decomposition and the dimensions derived from the THINGS behavioral odd-one-out experiment: the correlation matrix demonstrates that each neural dimension covaries with many of the behavioral dimensions, and that each behavioral dimension in turn covaries with many neural dimensions (Response Figure 4).

3. The following statement is too strong:

   The authors write: "our findings show that the shared representational dimensions of different brains can only be fully observed by hyperaligning the cortical activity patterns from one individual to another. This implies that the underlying dimensions of the representational code are not spatially localized but instead correspond to latent dimensions that are distributed."

   Another strong possibility that should be acknowledge here is that the anatomical alignment (MNI space) was insufficient to capture local distortions or warpings of anatomy across individuals. Cortical alignment based on folds, function, and other anatomical properties has been shown to yield much better alignments than MNI space does. Thus, rejecting the possibility for anatomical consistency of high dimensions seems premature.

We agree with the reviewer's characterization. It is certainly possible that more precise cortical alignment would better align the dimensions of visual representations across different individuals. We have added the following text to the manuscript to clarify this:

"However, we note that the anatomical alignment procedure used to align individuals to the standardized MNI space may not be optimal. More precise cortical alignment techniques based on cortical folding, function, and other anatomical properties such as cytoarchitectonics could potentially allow for the detection of more shared dimensions across subjects."

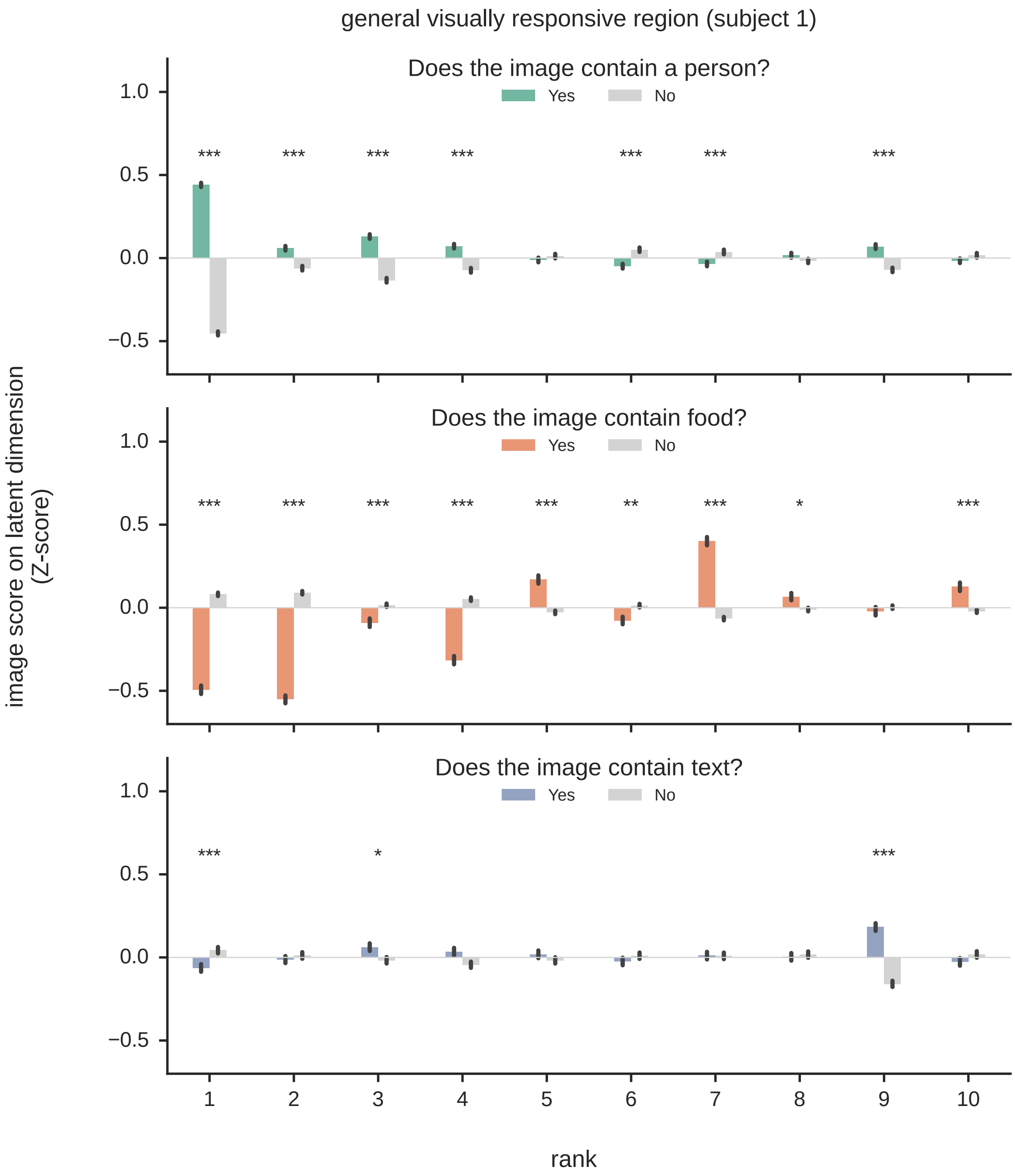

Response Figure 3: **Semantic category information is distributed over the low-rank dimensions of neural activity in the "general" visually responsive region of cortex.** Information about the presence of people (top), food (middle) and text (bottom) in an image is distributed over the first 10 latent dimensions of neural activity in the "general" visually responsive region of cortex, identified through within-subject cross-decomposition in an example subject (subject 1). Images that either contain (colored) or do not contain (gray) these object categories are projected onto each of these dimensions and these score distributions are compared using Mann-Whitney U tests to identify significant differences (*, $p < 0.01$; **, $p < 0.001$; ***, $p < 0.0001$). Bars and error bars indicate the means of the score distributions and their standard errors.

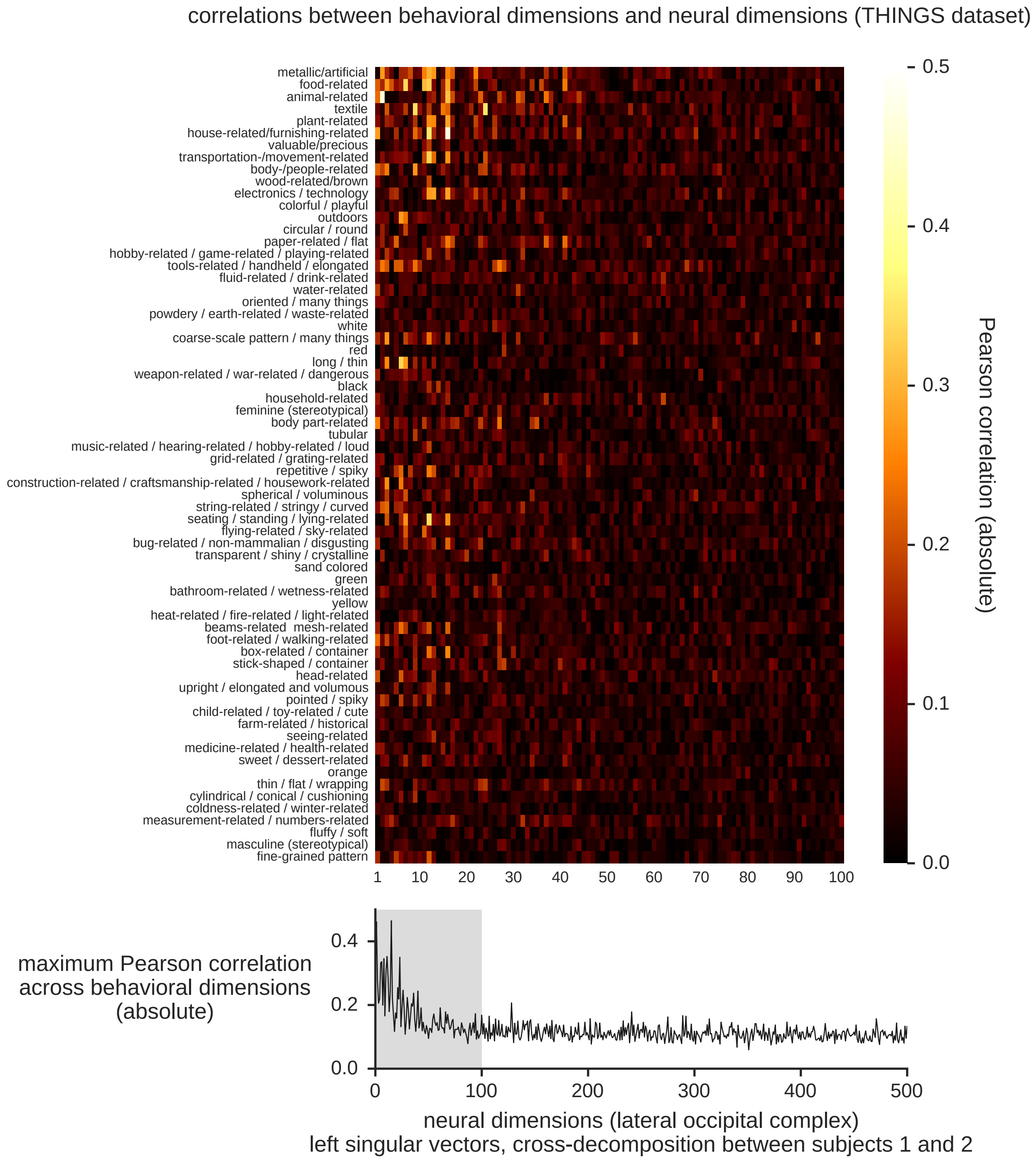

Response Figure 4: **Latent dimensions from the THINGS fMRI dataset are correlated with dimensions obtained from a large-scale behavioral experiment.** We perform between-subject cross-decomposition of the neural responses in the lateral occipital complex (LOC) from subjects 1 and 2 and project all the images onto the latent dimensions from subject 1. Then, we average these projections across all images within each of the 720 object categories and correlate each of these neural dimensions with each of the 66 interpretable dimensions that capture human object similarity judgments in a large-scale behavioral experiment (Hebart et al., 2023). (Top) We find correlations between the neural and behavioral dimensions but no evidence for a one-to-one correspondence between these dimensions. (Bottom) For each neural dimension, we compute the maximum absolute Pearson correlation with all behavioral dimensions and find that the correlations between neural and behavioral dimensions are strongest in the low-rank dimensions and fall off rapidly.

4. Richness of stimulus set

I think the way the authors have hedged to remove any discussion of the role of stimulus "richness" in estimating dimensionality is OK, but I do think it's obvious that stimulus richness must matter to estimates of dimensionality. To take an extreme case, a stimulus consisting of only black and white checkerboards flashed on and off would not be an effective stimulus for much of visual cortex and thus would not be likely to elicit the same dimensionality of responses as a large set of natural images. I think this is worth thinking about and potentially worth discussing, tho as I said the path the authors have taken is acceptable (if less interesting).

We agree with the reviewer—certainly, in extreme examples such as the proposed checkerboards, we would expect the dimensionality of neural responses to be lower. However, we note that in previous work, even fairly simple synthetic stimuli still evoked high-dimensional responses in mouse V1 (Figure 3 in Stringer et al. (2019)). Nonetheless, it is possible that such stimuli may evoke lower-dimensional representations in the later stages of visual processing in human visual cortex. We have added a paragraph discussing this to the manuscript:

"We note that this high-dimensional structure is observed when analyzing a rich, large-scale dataset containing neural responses to many thousands of natural images sampling a diverse range of scenes and objects. It is unclear to what extent this power-law covariance spectrum in neural responses depends on the diversity of the stimulus set. In mouse V1, even fairly simple synthetic stimuli still evoked high-dimensional responses (Figure 3 in Stringer et al. (2019)). Whether a less rich stimulus set would evoke the same power-law spectra in high-level human visual cortex remains an open question."

3 Reviewer #3

I thank the authors for their detailed rebuttal and extensive revisions. In particular, the Gabor-model analysis significantly strengthens the paper.

We thank the reviewer for their positive assessment.

All but two of the issues raised in my previous review have been sufficiently addressed. The two remaining issues are detailed below.

First, since statistical model comparisons between scale-free and alternative spectral distribution models were not conducted, the language regarding "scale-free" representations should be tempered. While straight lines on log-log plots are consistent with the authors' claim, the inferential results (i.e., the permutation tests) only indicate that the representations are high-dimensional, not necessarily scale-free. The claim of "scale-free" representations appears throughout the paper, including in the title, abstract, and main text.

We agree with the reviewer and have added a clear caveat to the Discussion section (under "Limitations of our analysis") in a paragraph titled "Alternative heavy-tailed distributions":

"While our results are consistent with scale-free neural data that obey a power law over many orders of magnitude—as observed from the linearity of the spectra on the log-log plots—there may be other heavy-tailed distributions that are also compatible with these data, such as lognormal distributions, or power-law distributions with an exponential cut-off. We remain agnostic to this possibility, but we emphasize that the unbounded scaling of our spectra with dataset size (Figure S2) suggests that we should not attempt to quantify the dimensionality with a single scalar value: there is reliable visual information distributed over all latent dimensions, up to the limit of detectability. Successfully distinguishing between multiple heavy-tailed distributions requires collecting enormous datasets to achieve high enough spectral resolution, especially with the binning procedure we use to extract signal in noisy subspaces. Indeed, similar results in the systems neuroscience literature also refer to such high-dimensional spectra as power-law spectra, though in principle other heavy-tailed distributions may also fit the data reasonably well (Manley et al., 2024; Stringer et al., 2019)."

Second, my comment from the previous review remains largely unaddressed:

> It is somewhat unclear which methodological components are novel and which are adapted from previous work. Clarifying this distinction is important not only for assigning appropriate credit but also for identifying which aspects of the analysis may require further validation, for example through simulation studies. In particular, it would be helpful to clarify whether the correlation coefficient defined in Equation 1, used to quantify representational similarity across latent dimensions, is based on prior work or whether it is introduced here for the first time.' '
>
> As it stands, the Methods section (and especially subsections 5.2–5.3) does not clearly delineate which methods are adapted from previous research (particularly Stringer et al., 2019) and which are novel. This clarification should be made in the paper itself, not in the rebuttal.
>
> Other than these two issues, I believe the paper is ready for publication.

We apologize for the omission of this clarification in the main manuscript. We have added a paragraph titled "Relationship to other spectral estimators" to the Methods section (under Subsection 5.2):

"Note that the first step of the cross-decomposition procedure is a well-established statistical method also known as Procrustes transformation or partial least squares singular value decomposition (PLS-SVD). This alignment of two datasets based on the spectral decomposition of their cross-covariance is also used in the hyperalignment procedure (Haxby et al., 2011), though Haxby et al. (2011) focus on aligning one dataset to another and not on the underlying spectrum of variance shared between the datasets. Stringer et al. (2019) developed cross-validated principal component analysis (cvPCA) to estimate such a spectrum of stimulus-related variance shared between repeated neural responses to the same stimuli. While the cross-decomposition approach shares similarities with the covariance eigenspectrum obtained by cross-validated PCA (cvPCA) (Stringer et al., 2019), it also departs from it in two important ways. First, cvPCA can only be applied to within-subject analyses. To explore the level of shared representations between subjects, we need a method that can functionally align their representations—as cross-decomposition does. Second, cvPCA examines reliability across stimulus repetitions for a set of images but does not examine generalization to new images. Because our goal is to characterize representational properties that generalize over images, our cross-decomposition approach assesses both reliability across stimulus repetitions *and* generalization to new stimuli. As a result of these differences, cross-decomposition and cvPCA characterize different statistical quantities whose spectra naturally differ. This is illustrated in Figure 1, where standard PCA, cvPCA, and cross-decomposition exhibit different spectral decays due to their different generalization requirements. Note that an important consequence of this is that the power-law exponents observed for different estimators should not be directly compared. Additionally, we introduce here a method for computing a between-subject spectral correlation coefficient, which characterizes the range of dimensions over which the representation in one subject is shared with another. This estimator is described in detail below."

References

Contier, O., Baker, C. I., & Hebart, M. N. (2024). Distributed representations of behaviour-derived object dimensions in the human visual system. *Nature Human Behaviour*. https://doi.org/10.1038/s41562-024-01980-y

Haxby, J. V., Guntupalli, J. S., Connolly, A. C., Halchenko, Y. O., Conroy, B. R., Gobbini, M. I., Hanke, M., & Ramadge, P. J. (2011). A common, high-dimensional model of the representational space in human ventral temporal cortex. *Neuron*, *72*(2), 404–416. https://doi.org/10.1016/j.neuron.2011.08.026

Hebart, M. N., Contier, O., Teichmann, L., Rockter, A. H., Zheng, C. Y., Kidder, A., Corriveau, A., Vaziri-Pashkam, M., & Baker, C. I. (2023). Things-data, a multimodal collection of large-scale datasets for investigating object representations in human brain and behavior. *eLife*, *12*. https://doi.org/10.7554/elife.82580

Khosla, M., Ratan Murty, N. A., & Kanwisher, N. (2022). A highly selective response to food in human visual cortex revealed by hypothesis-free voxel decomposition. *Current Biology*, *32*(19), 4159–4171.e9. https://doi.org/10.1016/j.cub.2022.08.009

Lin, T.-Y., Maire, M., Belongie, S., Bourdev, L., Girshick, R., Hays, J., Perona, P., Ramanan, D., Zitnick, C. L., & Dollár, P. (2014). Microsoft coco: Common objects in context. https://doi.org/10.48550/ARXIV.1405.0312

Manley, J., Lu, S., Barber, K., Demas, J., Kim, H., Meyer, D., Traub, F. M., & Vaziri, A. (2024). Simultaneous, cortex-wide dynamics of up to 1 million neurons reveal unbounded scaling of dimensionality with neuron number. *Neuron*, *112*(10), 1694–1709.e5. https://doi.org/10.1016/j.neuron.2024.02.011

Pachitariu, M., Stringer, C., Dipoppa, M., Schröder, S., Rossi, L. F., Dalgleish, H., Carandini, M., & Harris, K. D. (2016). Suite2p: Beyond 10, 000 neurons with standard two-photon microscopy. https://doi.org/10.1101/061507

Pachitariu, M., Stringer, C., & Harris, K. D. (2018). Robustness of spike deconvolution for neuronal calcium imaging. *The Journal of Neuroscience*, *38*(37), 7976–7985. https://doi.org/10.1523/jneurosci.3339-17.2018

Papale, P., Wang, F., Self, M. W., & Roelfsema, P. R. (2025). An extensive dataset of spiking activity to reveal the syntax of the ventral stream. *Neuron*, *113*(4), 539–553.e5. https://doi.org/10.1016/j.neuron.2024.12.003

Stringer, C., Pachitariu, M., Steinmetz, N., Carandini, M., & Harris, K. D. (2019). High-dimensional geometry of population responses in visual cortex. *Nature*, *571*(7765), 361–365. https://doi.org/10.1038/s41586-019-1346-5

Veit, A., Matera, T., Neumann, L., Matas, J., & Belongie, S. (2016). Coco-text: Dataset and benchmark for text detection and recognition in natural images. https://doi.org/10.48550/ARXIV.1601.07140
